# The evolutionary origin of jaw yaw in mammals

**DOI:** 10.1038/srep45094

**Published:** 2017-03-21

**Authors:** David M. Grossnickle

**Affiliations:** 1 University of Chicago, Committee on Evolutionary Biology, Chicago, 60637, USA

## Abstract

Theria comprises all but three living mammalian genera and is one of the most ecologically pervasive clades on Earth. Yet, the origin and early history of therians and their close relatives (i.e., cladotherians) remains surprisingly enigmatic. A critical biological function that can be compared among early mammal groups is mastication. Morphometrics and modeling analyses of the jaws of Mesozoic mammals indicate that cladotherians evolved musculoskeletal anatomies that increase mechanical advantage during jaw rotation around a dorsoventrally-oriented axis (i.e., yaw) while decreasing the mechanical advantage of jaw rotation around a mediolaterally-oriented axis (i.e., pitch). These changes parallel molar transformations in early cladotherians that indicate their chewing cycles included significant transverse movement, likely produced via yaw rotation. Thus, I hypothesize that cladotherian molar morphologies and musculoskeletal jaw anatomies evolved concurrently with increased yaw rotation of the jaw during chewing cycles. The increased transverse movement resulting from yaw rotation may have been a crucial evolutionary prerequisite for the functionally versatile tribosphenic molar morphology, which underlies the molars of all therians and is retained by many extant clades.

The evolution of mammals from pre-mammalian cynodonts was accompanied by significant changes to dentitions and musculoskeletal anatomies of jaws^1^,^2^,^3^,^4^,^5^,^6^,^7^,^8^,^9^,^10^. These include the appearance of diphyodonty^8^ (i.e., single dental replacement) and increased occlusal complexity^4^,^5^,^6^,^10^, suggestive of greater masticatory efficiency and precise control of jaw musculature. In addition, evolution of the jaw articulation between the dentary and squamosal resulted in the loss of load-bearing jaw joint functions for middle ear elements, possibly allowing for a greater diversity of jaw morphologies by permitting increased resultant forces at the jaw joint^11^,^12^. These evolutionary changes likely played a role in a taxonomic and morphological diversification of mammaliaforms in the Jurassic^5^,^13^,^14^,^15^,^16^ (Fig. 1), which included the origin of therians (i.e., eutherians and metatherians) and australosphenidans (including monotremes).

Therians and australosphenidans now comprise all modern mammals, and therians in particular have achieved considerable taxonomic, morphological, and ecological diversity. However, their origin and early history remains surprisingly enigmatic, due in large part to limited fossil evidence. Paleontological research on early therians often focuses on morphological transitions, phylogenetic relationships, and the timing of the clade’s origination and early diversification^17^,^18^,^19^,^20^,^21^,^22^,^23^,^24^,^25^. However, paleontological examinations of jaw biomechanics are lacking, and a better understanding of this aspect of therian biology may offer considerable insight into the early evolution of the clade.

Therians and australosphenidans evolved tribosphenic molar morphologies, likely through convergent evolution^13^,^22^,^26^,^27^,^28^,^29^ (although see Rich *et al*.^30^ for an opposing view). Evidence suggests that the evolution of the tribosphenic molar morphology was a critical development in mammalian history. For instance, tribosphenic molar occlusion is extremely precise and involves multiple shearing crests^19^,^22^,^31^,^32^,^33^,^34^,^35^, resulting in a system that seems especially effective for rapidly cutting chitinous exoskeletons of insects. The molars are also capable of crushing food matter in the talonid basin^32^,^35^ (Fig. 1), a function that is not apparent in earlier molar morphologies and may allow for a broad diversity of diets. The functional significance of the morphology is supported by the continued prevalence of tribosphenic (or tribosphenic-like) molars in many modern mammal groups (e.g., microchiropterans, didelphids, dasyurids, scandentians, and many eulipotyphlans), despite evolving at least 160 million years ago (Ma)^17^,^27^.

An essential step in the evolution of the tribosphenic molar morphology of therians was the appearance of a talonid shelf in the lower molars of stem cladotherians (i.e., “eupantotherians”)^1^,^2^,^18^,^19^ (Fig. 1). The shelf acts as an extended shearing surface for the paracone of the upper molar. In tribosphenic molars the talonid shelf expands into the talonid basin and has a crushing function^1^,^2^,^19^,^22^,^32^,^35^. Stem cladotherians (comprised primarily of Dryolestida, Amphitheriida, and “peramurans”) were abundant in the Late Jurassic and regionally diverse in South America in the Cretaceous. Together with therians they form Cladotheria. Thus, cladotherians have been globally diverse for over 150 million years, and examining the early history of the clade is critical to understanding the origins of modern mammalian diversity.

The earliest cladotherians evolved notable morphological changes to molars, jaws, and ears. These include:

1. Molars with a talonid shelf, an evolutionary precursor to the talonid basin of tribosphenic molar morphologies (Fig. 1).
2. A prominent, posteriorly positioned angular process (AP) of the mandible^14^,^36^,^37^.
3. The potential loss of a bony attachment between the middle ear elements and jaw. Early crown mammals such as eutriconodontans and spalacotherioids often possess a bony connection between the middle ear and jaw via an ossified Meckel’s cartilage^23^,^38^,^39^,^40^,^41^ (Fig. 1), but this connection does not appear to be present in cladotherians. (However, mandibles of early cladotherians often possess a Meckel’s groove^20^,^42^,^43^,^44^, indicating that an ear-jaw connection may be maintained by cartilage.)

In light of these morphological changes, this study has two goals. The first is to use morphometrics to quantify the morphological changes to the jaw processes in early mammal groups, with a focus on comparing early cladotherians to closely related clades. The second goal is to model the changes to the jaw muscle vectors that are expected to accompany the morphological changes, allowing the functional significance of musculoskeletal changes in cladotherians to be assessed. The superficial masseter (SM) and medial pterygoid (MP) are two of the major masticatory muscles and they insert on the AP (Fig. 2), which shows considerable morphological variation among early mammal groups^14^. Thus, changes to the force vectors of these muscles are the focus of the functional analyses, although the temporalis muscle (TM) is also incorporated. Results of these analyses are considered in light of concurrent morphological and functional changes to the molars, with special focus on the evolution of the tribosphenic molar morphology.

Based on results of the morphometrics and functional analyses, I develop a novel hypothesis for the simultaneous origin of unique jaw, dental, and ear characters in cladotherians. Central to this hypothesis is the observation that a majority of modern and extinct mammals, including early cladotherians, possess chewing cycles with substantial transverse movement of the molars^35^,^45^,^46^,^47^,^48^,^49^,^50^,^51^,^52^,^53^,^54^,^55^,^56^,^57^,^58^,^59^,^60^,^61^,^62^. This includes taxa with tribosphenic molar morphologies, which appear to require mediolateral molar movement for extended shearing and crushing functions^2^,^35^,^48^,^49^,^50^,^51^,^52^. There are two means of producing transverse molar movement: (i) mediolateral translation of the jaw (along the *z* axis of Fig. 2) and (ii) yaw rotation of the jaw around a dorsoventrally oriented axis (Fig. 2D). However, considerable evidence indicates that yaw is the primary means of producing transverse molar movement during occlusion in modern and fossil therians, with studies demonstrating yaw in didelphids, diprotodontians, eulipotyphlans, scandentians, *Solenodon*, suids, cervids, and primates^45^,^46^,^47^,^48^,^49^,^50^,^53^,^54^,^55^,^56^,^57^,^58^,^59^,^61^. For instance, primates and tree shrews with tribosphenic (or tribosphenic-like) molar morphologies have been described as having two phases of occlusion that both involve yaw rotation^52^,^55^. In contrast, transverse movement produced from mediolateral translation along the *z* axis during occlusion may be considerably less common in mammals, with jaw movements in carnivorans being a notable exception^62^,^63^.

Yaw rotation is produced by asynchronous contractions of jaw muscle groups^45^,^54^,^56^,^57^,^58^,^59^,^61^. For instance, concurrent peak contractions of the balancing-side (i.e., non-chewing side) MP, balancing-side SM, and working-side (i.e., chewing side) temporalis muscle (TM) during the fast close portion of the chewing cycle causes the working-side jaw to move laterally via yaw. The three muscles involved in this movement (Fig. 2) were termed the Triplet I muscle group by Weijs^56^. The fast close is followed by the power stroke stage of the chewing cycle in which molars are in occlusion and the complementary Triplet II muscles (i.e., working-side MP, working-side SM, and balancing-side TM) reach peak contraction, rotating the working-side jaw medially.

Although considerable variation in muscle activity exists, the asynchronous but coordinated contractions of Triplet muscle groups are common among extant mammals and are believed to be a primitive trait of therians^45^,^56^,^59^. However, the origin of Triplet muscle activity has not been investigated using fossil data, and it has yet to be determined whether Triplet muscle groups were present in stem therian lineages. Further, previous studies on jaw biomechanics and mammalian origins have largely focused on pre-mammalian lineages rather than early crown mammals^11^,^12^,^64^. Thus, musculoskeletal changes seen in early cladotherians may offer new insight into the evolutionary origin of the chewing cycles that are observed in modern mammals.

## Results and Discussion

### Angular process (AP) shape

Results of a geometric morphometric analysis of AP shape suggest that a posterior, prominent AP is a derived trait of Cladotheria (Fig. 3). Relative to APs of stem mammaliaforms, the APs of cladotherians are much more posterior in position. This is especially true for dryolestoids, which explains why they occupy a unique region of morphospace (i.e., negative PC1 values in Fig. 3). The prominent AP of early cladotherians is especially noteworthy because a distinct AP is not present in closely related spalacotherioids, eutriconodontans, and multituberculates (Fig. 3). Based on the phylogenetic positions of these clades, the results support previous suggestions that the APs of non-mammalian cynodonts (which have been referred to as “pseudangular” processes) are not homologous to the APs of therians^18^,^65^,^66^ (but see Rougier *et al*.^67^ and Abdala and Damiani^68^ for an opposing view).

**Figure 1:**
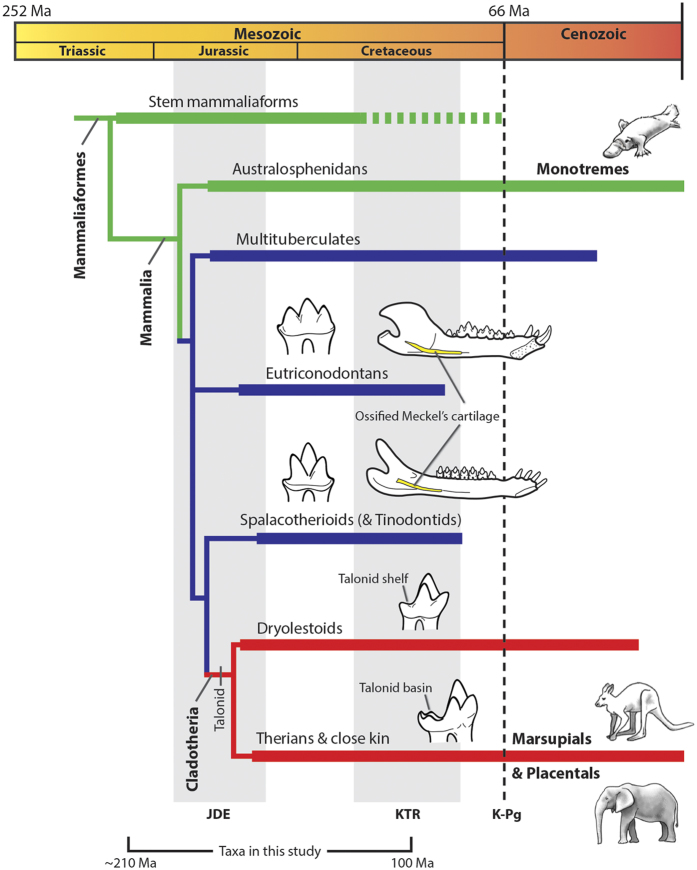
Phylogeny of early mammaliaforms^26^. Marsupials and placentals are crown therians, and monotremes are crown australosphenidans. The branch colors are based on morphological similarities of the mandibles (see below). A Jurassic diversification event (JDE) resulted in the origination of many Mesozoic mammalian lineages^13^,^15^,^16^, and the Cretaceous Terrestrial Revolution (KTR) included a taxonomic turnover of mammalian faunas^14^,^78^,^85^. The eutriconodontan jaw is *Yanoconodon*^38^, the spalacotherioid jaw is *Maotherium*^39^, and mammal images are courtesy of April Neander. Abbreviations: K-Pg, Cretaceous-Paleogene boundary; Ma, millions of years ago.

In addition to the posteriorly positioned AP of jaws, the talonid shelf of lower molars is a character that appears at the cladotherian node^26^,^29^(Fig. 1). Thus, the prominent AP of jaws and the talonid shelf of molars seem to have evolved concurrently in the earliest cladotherians.

### Jaw joint and coronoid process elevation

The articular surface of the condylar process (i.e., jaw joint surface) is considerably elevated in dryolestoids (Fig. 4A). Spalacotherioids and therians (and close kin) also have moderately elevated jaw joints, relative to the depressed jaw joints of eutriconodontans and multituberculates. In comparison to the jaw joint, the differences in coronoid process elevations among early mammal groups are not as distinct (Fig. 4B). The average (median) elevation above the molar row for all groups is between 21.5% and 25.5% of the length of the jaw, with the greatest range of values in stem mammaliaforms.

### Pitch and roll

**Figure 2:**
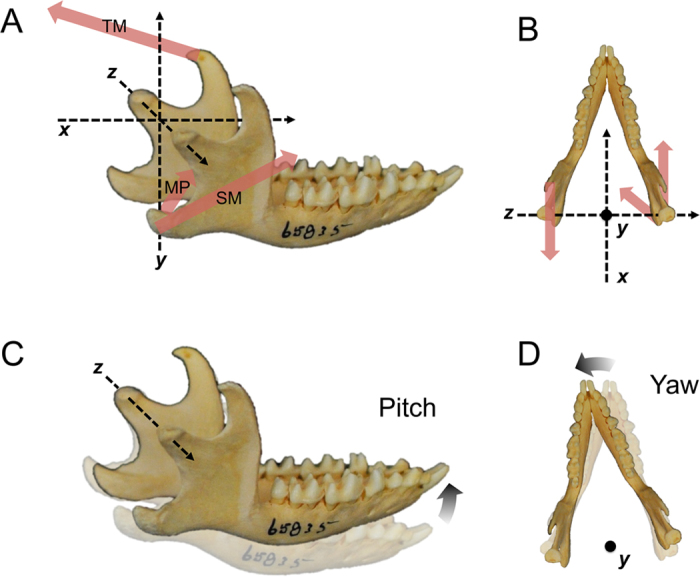
(**A,B**) The *x, y*, and *z* coordinate planes used in this study, displayed on a mandible in oblique lateral view (**A**) and dorsal view (**B**). The red arrows represent the approximate force vectors of a Triplet muscle group, which contract concurrently in many modern mammals^45^. This group includes the medial pterygoid (MP) and superficial masseter (SM) muscles of one hemimandible, and the temporalis muscle (TM) of the opposing hemimandible. (**C**) Pitch rotation around a mediolaterally oriented (*z*) axis through the condylar processes. (**D**) Yaw rotation around a dorsoventrally oriented (*y*) axis. (The axes of rotation in (**C** and **D**) are arbitrarily positioned to demonstrate potential jaw movements.) The mandible is of a hedgehog (*Atelerix*) from the Field Museum of Natural History (FMNH 65835).

To examine functional changes in therians and their close ancestors, three-dimensional (3D) jaw models were constructed for eutriconodontans, spalacotherioids, dryolestoids, and early therians (and close kin) (Figs 5 and 6, Supplementary Fig. S3). Jaw joint and coronoid process elevations in the models are based on the median values for mammal groups in Fig. 4. Since the relative elevation of the coronoid process does not vary considerably among the mammal groups (Fig. 4b, Supplementary Table S3), the model images are simplified by keeping the TM insertion location (purple point in Fig. 6) and vector (purple arrow in Fig. 6) constant in the jaw model. However, the slight differences in the height of the coronoid are included in calculations of moment values. The expected muscle insertion sites for the MP and SM (Figs 5 and 6) are based on mean shapes of the AP region from Fig. 2 and locations of fossae in the jaws. Due to a scarcity of preserved 3D skulls of early mammals, locations of muscle origins are uncertain and are based primarily on average measurements of modern mammal analogs (Supplementary Table S2). Origin locations are kept constant in the models shown in Fig. 6, but the Supplementary information includes additional analyses that examine variation associated with potential evolutionary changes to these positions.

For pitch rotation of the jaw, results indicate that moment (i.e., moment of force, or torque) values decrease with the evolution of the prominent, posterior AP of early cladotherians (Fig. 6A). This pattern remains when various force values are assigned to the muscles (see Methods; Fig. 6) and when analyses are repeated using different muscle origin locations (Supplementary Fig. S5). Moment values for therians are slightly greater than for dryolestoids but remain less than the values of eutriconodontans and spalacotherioids.

For roll rotation of a hemimandible, moment values are small for all mammal groups and do not show a distinct trend (Fig. 6B). The values are likely low because the relatively large balancing-side TM is not involved in the calculations (since only the working-side hemimandible is tested; Fig. 6), and because the MP force vector counteracts the SM force vector and works against roll (in the direction tested here). This is consistent with the observation by Crompton^6^ that there is minimal roll of the working-side hemimandible in *Didelphis* during the power stroke of the chewing cycle (although roll is significant during additional stages of the chewing cycle), with the MP helping to stabilize the working-side hemimandible against roll during this stage.

### Yaw

Unlike results for pitch and roll, the moment values for yaw were greater for early cladotherians than for eutriconodontans or spalacotherioids (Fig. 6). This is especially apparent when the axis of rotation is near the balancing-side jaw joint, as moments are approximately 50% greater for dryolestoids than eutriconodontans. The results for therians (and close kin) are slightly less in value than those of dryolestoids, but they are greater than those of eutriconodontans and spalacotherioids. These results suggest that the posterior AP evolved in response to selection for increased mechanical advantage during yaw rotation. The greater mechanical advantage may be beneficial during both the fast close and power stroke stages of the chewing cycle, since yaw typically occurs during both stages^46^,^47^,^48^,^49^,^53^,^57^,^59^.

The results for jaw yaw in dryolestoids are consistent with molar morphologies and wear patterns, which provide evidence for significant mediolateral movement during occlusion^35^,^51^,^52^,^60^,^69^. For instance, microwear scratches are often at a 35° angle relative to horizontal in dryolestoids^69^, and the talonid shelf surface that occludes with the paracone of the upper molar is at a similar angle (Fig. 5). Thus, lower molars of dryolestoids must include considerable medial movement during occlusion, which is most likely produced by yaw (based on evidence from additional mammal groups^45^,^46^,^47^,^48^,^49^,^50^,^53^,^54^,^55^,^56^,^57^,^58^,^59^,^61^). This provides support for the conclusion that cladotherian jaw and molar morphologies evolved in concert with additional yaw rotation.

During occlusion, molar morphologies may influence the specific directional path of molars and jaws^63^,^69^,^70^,^71^, and this could suggest that coordinated muscle activity may not be necessary for production of yaw rotation. However, even if molar morphologies direct the medial jaw movement during occlusion, this movement necessitates opposing lateral movement via yaw during the fast close stage of the subsequent chewing cycle to re-align the molars for occlusion. Further, muscle activity is likely required to redirect the molars from a dorsolateral movement during the fast close stage of the chewing cycle to a dorsomedial movement at the onset of the power stroke stage (in which the molars have occluded), thus initiating the power stroke but not necessarily controlling precise movement during occlusion. Finally, studies of modern pigs and primates demonstrate yaw rotation (produced via Triplet muscle groups) during occlusion even though their bunodont molar morphologies do not passively direct the movement^57^,^61^. Thus, these taxa represent examples in which jaw muscles (and not molar morphologies) must be initiating yaw rotation during mastication.

**Figure 3:**
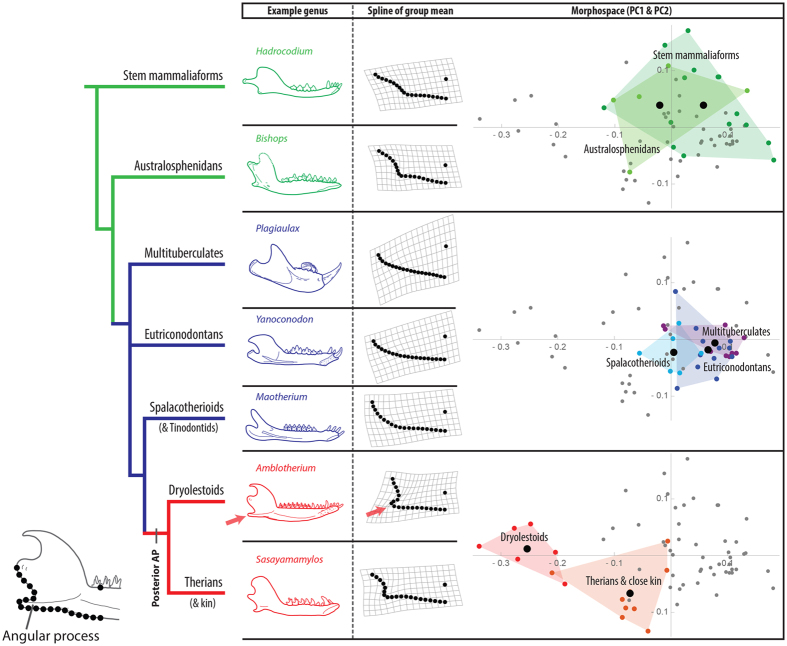
Geometric morphometric analysis of angular process (AP) shapes for mammaliaform and mammalian genera from the latest Triassic through Early Cretaceous (i.e., ~210–100.5 Ma). ‘Green’ lineages possess an anteriorly positioned AP. The ‘blue’ lineages are clades that do not possess a distinctive AP. A posterior, prominent AP appears in cladotherians (‘red’ lineages) and is highlighted by red arrows. Thin plate splines for each group show the mean shape of the APs. The three principal component analysis (PCA) morphospace plots (right) are replicates of the same plot, but in each replicate different mammal groups of the phylogeny are designated by polygons. Black points in the polygons represent the mean AP shape for each group. The horizontal axis is PC1 (54.5% of variance), and the vertical axis is PC2 (17.4% of variance). See Supplementary Figure S4 for labeled versions of the PCA plots. The *Amblotherium* jaw is after Simpson^86^, and sources for additional jaw images are provided in Supplementary Table S1.

Spalacotherioids may represent morphological and functional intermediates between eutriconodontans and early cladotherians. They have moment values that are between those of eutriconodonts and cladotherians for the three types of rotation (Fig. 6). Spalacotherioid molar morphologies suggest that their occlusion includes more mediolateral movement than that of eutriconodontans^51^ (Fig. 5), and their jaws possess a posteroventral ‘bulge’ that may alter muscle vectors in a similar manner as an angular process (Figs 3 and 5). However, the lack of a talonid suggests that the transverse movement of lower molars in spalacotherioids is not as extensive as that seen in dryolestids and early therians.

Recent fossil discoveries suggest that eutriconodontans and spalacotherioids possess an ossified Meckel’s cartilage that connects the lower jaw and middle ear^23^,^38^,^39^,^40^,^41^. Although a Meckel’s groove is present in some early cladotherians^20^,^42^,^43^,^44^, no cladotherian fossils have been discovered with an ossified Meckel’s cartilage. Thus, it is likely that an ear-jaw connection in early cladotherians was either not present (at least in adults) or was maintained by cartilaginous tissue rather than bone. If this is the case, it represents an additional morphological change that may have evolved concurrently with greater yaw rotation of the jaw. In jaws with attached middle ears, yaw might create additional stress on attached middle ear elements because, unlike pitch, it involves protraction and retraction of hemimandibles at the condyles (Fig. 2), likely resulting in tension and compression for attached ear elements. If there is considerable flexibility at the mandibular symphysis or physical restrictions at the glenoid fossae, some retraction and protraction may be replaced by *z* axis translation of hemimandibles, but this movement is also expected to result in stress on middle ear elements. Therefore, the lack of a rigid ear-jaw connection in cladotherians may have allowed greater jaw mobility and decreased the amount of strain on the ear during yaw rotation.

### Therians and australosphenidans

**Figure 4:**
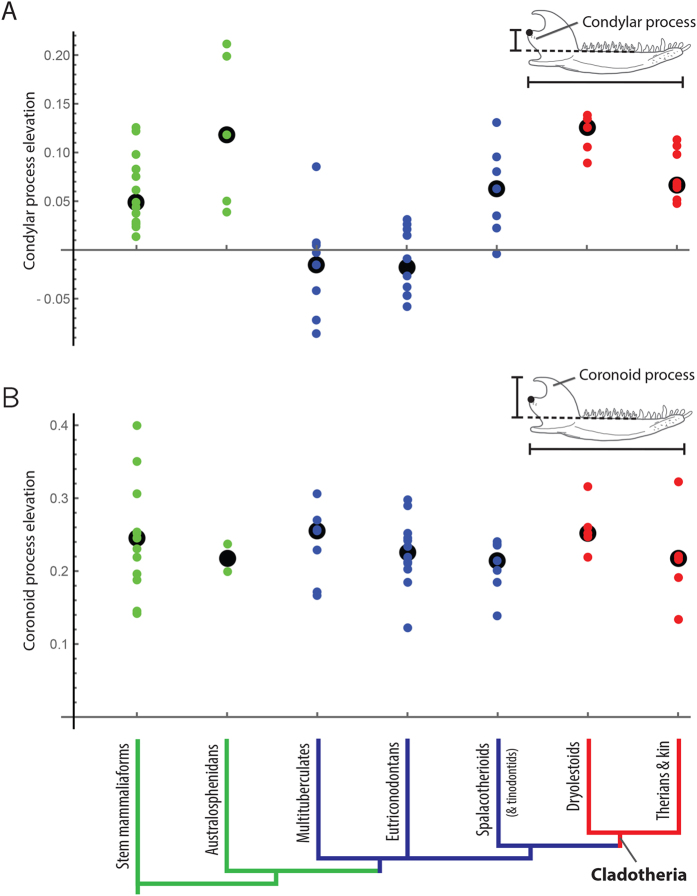
Condylar process (i.e., jaw joint) elevation (**A**) and coronoid process elevation (**B**) for mammaliaform genera, measured as the elevation (or depression) from the alveolar margin (dashed line) and standardized by dividing by jaw length. Large, black points represent the median value for each group, and these values are used in the jaw models and moment calculations. See Supplementary Table S3 for individual results.

**Figure 5:**
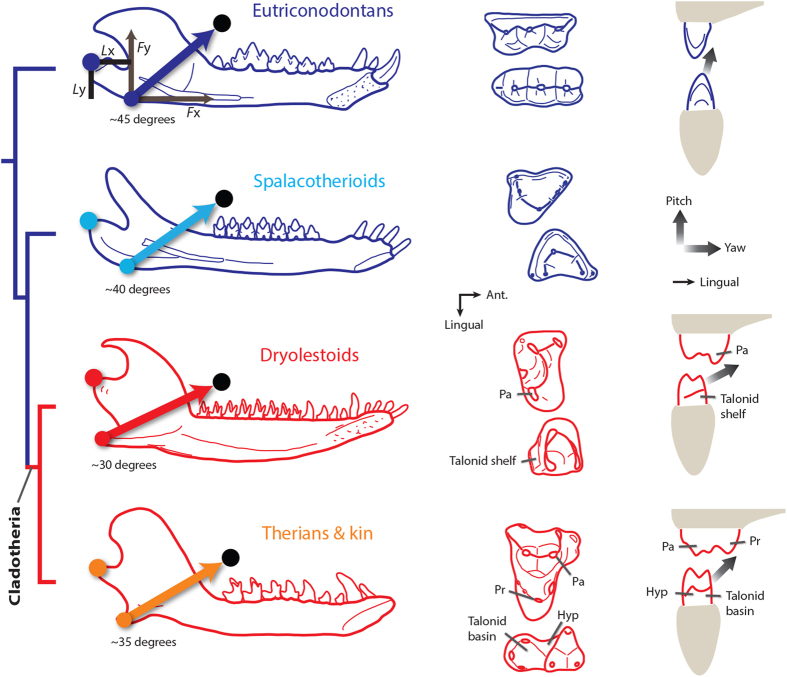
Representative jaws and molars of early cladotherians and their close relatives. Arrows signify the superficial masseter (SM) muscle, and black points represent the predicted muscle origin location. The approximate angles of the SM force vector (relative to the molar row) are given, and it is predicted that this angle decreased with the evolution of the posterior AP of cladotherians. As an example to help illustrate moment calculations (see Methods), the eutriconodontan jaw image includes the 2D *x* and *y* force components (*F*x and *F*y; scaled to muscle length for simplicity) and corresponding moment arms (*L*y and *L*x, respectively) for the SM. Representative upper and lower molars are shown in occlusal view. On the far right are schematic illustrations of lower molar movement during occlusion in eutriconodontans, dryolestoids, and therians^52^. Eutriconodontan occlusion is largely orthal in direction and cladotherian occlusion includes considerable medial movement via yaw as the paracone (Pa) occludes with the talonid shelf or hypoflexid (Hyp). The protocone (Pr) of therian tribosphenic molars occludes with the talonid basin. Sources for jaw images are given in Supplementary Table S1. From top to bottom, the molar images are *Priacodon*^87^, *Spalacotherium*^4^, *Dryolestes*^37^, and *Prokennalestes*^20^.

**Figure 6:**
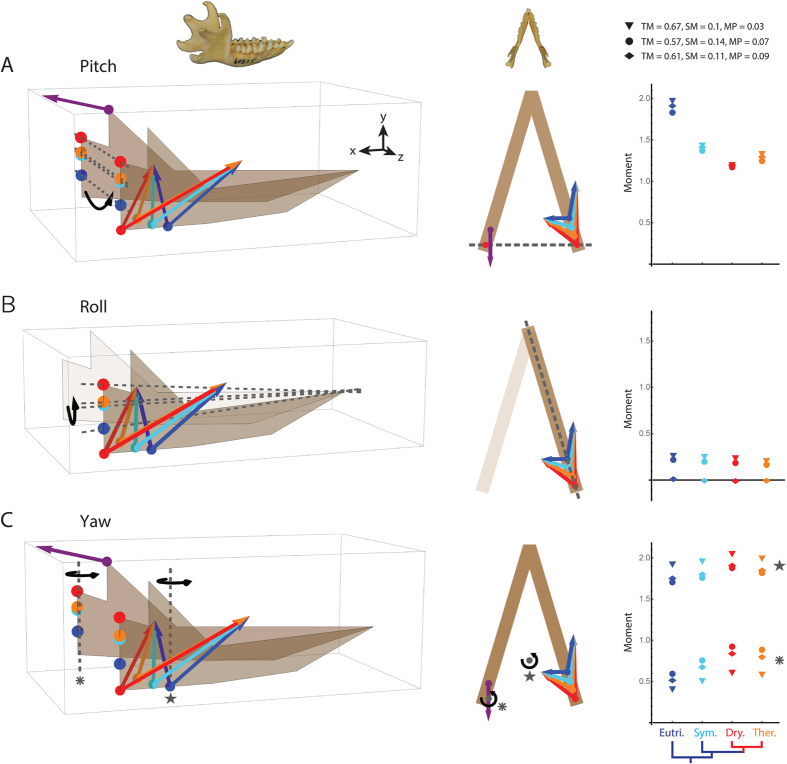
3D jaw models in oblique lateral view (left) and dorsal view (center), and moment (i.e., torque) values for musculoskeletal configurations of the mammal groups (right). Locations of the jaw joint and muscle insertions for each group are inferred from average results of the morphometric analyses (Figs 3 and 4, Supplementary Table S3). See Fig. 2 for muscle labels and Supplementary Figure S3 for point coordinates. Arrows represent the direction of the muscle forces (the lengths do not reflect the magnitude of the force vectors), with SM and MP vectors ending at the expected muscle origins but the TM vector is truncated. For simplicity, the TM vector (purple arrow) and coronoid process elevation are kept constant among groups in this figure, but slight changes in coronoid elevation among groups (Supplementary Table S3) are incorporated in calculations of moment values. Color assignments: blue, eutriconodontans (Eutri.); cyan, spalacotherioids (Spalac.); red, dryolestoids (Dry.); and, orange, therians and close kin (Ther.). Dashed lines represent axes of rotation and black arrows denote the direction of rotation. (**A**) Models and moment calculations for pitch rotation around mediolaterally oriented (*z*) axes through both jaw joints. (**B**) Models and moment calculations for roll rotation around axes through the jaw joint and mandibular symphysis. (**C**) Models and moment calculations for yaw rotation around two dorsoventrally oriented (*y*) axes, which are matched with moment results for each axis by corresponding gray stars. All moment calculations were repeated for three different force vector assignments for the jaw muscles (see key and Methods).

In comparison to dryolestoids, the average jaw morphology of early therians (and close kin) includes an AP that is not as posteriorly positioned and a jaw joint that is not as elevated (Figs 3 and 4). Due to these differences, yaw moment values for therians are not as great as those for dryolestoids (Fig. 6). Based on the hypothesized correlation between jaw morphology and yaw rotation, this suggests that therian chewing cycles include less mediolateral movement via yaw than the chewing cycles of dryolestoids. This prediction is corroborated by evidence from molar morphologies. In the tribosphenic molars of therians, the talonid shelf expands into a talonid basin (Figs 1 and 5). The extended shearing groove of the talonid shelf (i.e., the hypoflexid groove) is reduced in size and its slope is often vertically steeper in tribosphenic molars of early therians^22^,^31^,^35^,^52^, indicating that molar movement is more dorsally oriented (and likely involves less transverse movement via yaw) in early therians than in dryolestoids (Fig. 5). Further, occlusal contact between the protocone and hypoconid of the talonid basin in tribosphenic molars may truncate the transverse movement during occlusion^35^. Thus, molar and jaw morphologies of therians appear to be consistent with the hypothesis that AP position (and possibly jaw joint elevation) is correlated with the amount of mediolateral movement during mastication.

Modern mammalian clades tend to possess derived musculoskeletal jaw anatomies and functions, meaning that analogous comparisons to early mammal groups should be made with caution. However, a couple similarities are worth noting. Tenrecs, *Solenodon*, and several extinct therian lineages possess zalambdodont molars that are morphologically analogous to dryolestoid molars in possessing a talonid shelf instead of a talonid basin^72^,^73^,^74^,^75^, and these taxa are believed to produce transverse molar movement via jaw yaw^48^,^49^. Consistent with dryolestoids and the predictions of this study, zalambdodont taxa possess APs that are positioned strongly posteriorly^72^,^74^,^75^. In contrast to zalambdodont taxa, the occlusion in most carnivorans is primarily dorsoventrally oriented (i.e., orthal) with little transverse movement^56^,^60^,^70^,^71^, although mediolateral translation along the *z* axis may occur^62^,^63^. Yaw in many carnivorans is also likely limited by the wrapping of the glenoid fossa around the condyle of the jaw, creating a hinge-like joint that does not permit the protraction and retraction of condylar processes that occurs during yaw (Fig. 2). Consistent with the predictions of this study, carnivorans typically possess a reduced AP and depressed jaw joint^14^,^47^. These traits are analogous to those of eutriconodontans, which include carnivorous taxa such as *Repenomamus*^76^, and likely improve mechanical advantage for pitch rather than yaw. The musculoskeletal anatomies of carnivorans and eutriconodontans may also permit greater bite force during wide gape (see Supplementary information).

Evidence suggests that australosphenidans and therians convergently evolved similar morphological changes to the jaws, molars, and ears. This includes the appearance of a tribosphenic molar morphology and loss of a rigid attachment between the middle ear and jaw^23^,^27^,^28^,^77^. Australosphenidans possess AP morphologies that are similar to those of therians (Fig. 3), and some taxa have elevated jaw joints like those of early cladotherians (Fig. 4). These convergences suggest selective pressures for similar functional morphologies, and they may provide an additional line of evidence for the hypothesized functional link between these changes and increased yaw rotation during mastication. However, the scarcity of australosphenidan fossils and questions concerning their phylogenetic affinity^28^,^29^,^30^ prohibit further conclusions regarding this group. See the Supplementary information for additional discussion of australosphenidans, as well as consideration of docodonts that possess tribosphenic-like molar morphologies.

### Triplet muscle groups

In the jaw models, the Triplet II muscles (Fig. 2) were chosen for moment calculations since they contract concurrently during the power stroke of the chewing cycle (i.e., during occlusion) in many modern mammal groups^45^,^56^,^59^. Using these muscles for calculations assumes that the early mammal clades have asynchronously contracting Triplet muscle groups in which the balancing-side TM (rather than the working-side TM) is contracting with the working-side SM and MP. However, this assumption is unlikely to affect results for pitch and roll. For instance, using the working-side TM instead of the balancing-side TM for calculations would result in the same values for pitch because the moment arm length would be identical. In addition, if the working-side TM was included in the calculations for roll, it would likely have little effect because the vector is largely parallel to the axis of rotation (resulting in very short moment arms). Thus, if the asynchronous contractions of Triplet muscles were not present in early mammal groups, it is unlikely that revised calculations would alter the broad patterns seen here for pitch and roll.

Unlike the results for pitch and roll, the results for yaw are largely dependent on the asynchronous contractions of the Triplet muscle groups. For instance, if the working-side TM (rather than balancing-side TM) contracted with the working-side SM and MP, then the posteriorly directed force vector would counteract the SM and MP vectors, and yaw rotation in the medial direction would be unlikely to even occur. Thus, the increased moment values for early cladotherians during yaw are reliant on the asynchronous contractions of the Triplet muscle groups. The importance of efficient yaw rotation during the power stroke of occlusion in cladotherians implies that the Triplet muscle groups evolved no later than the origination of this clade.

### Tribosphenic molar evolution

Medial movement of the working-side hemimandible was a likely prerequisite for the evolution of tribosphenic molars. It allows for extended contact between the protocone and the talonid basin, as well as the hypoflexid and paracone, during the power stroke stage of a chewing cycle^35^ (Fig. 5). Considerable evidence suggests that this medial movement of the working-side jaw is produced via yaw rotation (rather than *z* axis translation) in early therians^45^,^46^,^47^,^48^,^49^,^50^,^53^,^54^,^55^,^56^,^57^,^58^,^59^,^61^, especially since yaw rotation occurs in modern and fossil taxa with tribosphenic (or tribosphenic-like) molars^48^,^49^,^50^,^53^,^55^,^59^. Thus, yaw appears to be a particularly important component of tribosphenic molar occlusion, and increased yaw may have been a critical early step in the evolution of tribosphenic molars.

The additional mediolateral movement via yaw in early cladotherians could have aided taxa by increasing the amount of shearing per chewing cycle. Not only are the primitive trigonid shearing crests (i.e., those of spalacotherioid molars) maintained in early cladotherians, but the novel talonid also permits extended shearing^35^ (Fig. 5). Crompton^19^ and Davis^22^ document an increased number of wear facets on molars in cladotherians relative to stem mammaliaforms, suggesting increased occlusal complexity and precision.

Many mammalian groups diversified taxonomically and ecomorphologically in the Jurassic^13^,^14^,^15^,^16^, but therians were one of the few groups to survive the ecological and taxonomic turnovers of the Cretaceous Terrestrial Revolution (KTR) and Cretaceous-Paleogene (K-Pg) extinction event^12^,^13^,^14^,^15^,^16^,^26^,^78^ (Fig. 1). The differential survival and subsequent diversification of therians^13^,^25^ hints at a potential functional advantage for lineages with tribosphenic molars. For instance, by allowing crushing of food items such as plant matter and soft insect parts, the tribosphenic molar morphology probably assisted in broadening the dietary diversity of early therians. In turn, the dietary versatility and efficiency of therian molars may have been critical for the survival of the clade during its early history.

## Conclusions

An improved understanding of the early evolution and biology of Theria will help elucidate the origins of modern mammalian diversity. Here, I examine concurrent evolutionary changes to functional anatomies of jaws, molars, and ears in early cladotherian mammals, and I posit that these changes are associated with increased transverse movement via yaw rotation during chewing cycles. The appearance of the talonid shelf of molars in stem cladotherians (e.g., dryolestoids) likely assisted in medial movement during occlusion and acted as an extended shearing surface (Figs 1 and 5). Further, a posteriorly positioned AP may have evolved due to selection for muscle force vectors that produce greater mechanical advantages during yaw (Fig. 6). Finally, the potential loss of a rigid connection between the jaw and middle ear in early cladotherians might have resulted in fewer restrictions on mediolateral movement of the mandible. The jaws, molars and ears of australosphenidans (which include monotremes) are morphologically similar to those of therians, suggesting convergent evolution of similar functional traits in this group.

I hypothesize that these morphological and functional changes were a critical step in the evolutionary origin of the therian feeding system, which includes the tribosphenic molar morphology and Triplet muscle groups. To produce yaw rotation, the chewing cycle of modern therian mammals includes asynchronous contractions of Triplet I and Triplet II muscle groups (Fig. 2). Thus, the fossil evidence for increased yaw rotation in early cladotherians suggests that the Triplet system evolved no later than the origination of Cladotheria. In addition, increased mediolateral jaw movement may have been a prerequisite for the evolution of the functionally diverse and efficient tribosphenic molar morphology in therians (and possibly australosphenidans), and this movement was likely produced via yaw rotation. The continued presence of tribosphenic molars in many modern mammalian lineages provides strong evidence of its evolutionary importance. Thus, the concurrent evolutionary changes to jaws, molars, ears, and chewing cycles in early cladotherians may have been an especially significant event in mammalian evolution.

## Methods

### Mammal groups

The phylogeny of early mammals (Fig. 1) is based on Martin *et al*.^26^. The ‘therians and close kin’ group (i.e., Zatheria + *Amphitherium*) is monophyletic and is represented in this study by early therians, two peramurans (i.e., *Peramus* and *Tendagurutherium*), and *Amphitherium*. Two tinodontids (*Yermakia* and *Tinodon*) were included with the spalacotherioid group and one shuotheriid (*Pseudotribos*) was included with the australosphenidan group. The stem mammaliaforms group is paraphyletic and includes *Sinoconodon*, morganucodontids, *Hadrocodium*, docodonts and haramiyids. See the Supplementary information for additional details on the mammal groups.

### Morphometrics

The morphology of the posteroventral region of the dentary, which includes the AP when present, was quantified by collecting two-dimensional (2D) outlines using jaw images of fossil taxa from the literature (Supplementary Table S1). The sample includes 64 mammaliaform genera from the latest Triassic through Early Cretaceous (i.e., ~210–100.5 Ma). Using Wolfram’s Mathematica and Polly’s Geometric Morphometrics for Mathematica^79^, 20 equally spaced semilandmarks were placed along the outer margin of the jaw, starting at the posteroventral-most edge of the articulation surface of the condylar process (i.e., jaw joint) and ending at the location that is directly ventral to the point between the ultimate and penultimate molars (Fig. 3). A single landmark was placed on the dorsal margin of the jaw between the ultimate and penultimate molars (Fig. 3). The coordinates of the 20 semilandmarks and one landmark were subjected to geometric morphometric procedures^79^,^80^,^81^,^82^, which include a Procrustes superimposition^82^ and ordination using a principal components analysis. See the Supplementary information for additional details, including an explanation as to why sliding semilandmarks were not used in this analysis.

Condylar process elevation was measured as the elevation (or depression) of the jaw joint from the base of the molar row (i.e., alveolar margin). For genera with an extensive articulation surface (e.g., multituberculates), I chose the midpoint of the articulation surface as the jaw joint position. Coronoid process elevation was measured as the maximum elevation of the coronoid process from the base of the molar row. I standardized all measurements by dividing by jaw length (Supplementary Table S3). Sample sizes vary among the morphometric analyses due to the lack of preservation of some jaw processes in certain fossil specimens.

### Jaw biomechanics

I created 3D models of jaws using Wolfram’s Mathematica to calculate the moment (i.e., moment of force, or torque) for various musculoskeletal configurations. The modeling analyses build upon concepts and methods in previous biomechanical analyses of synapsid jaw evolution^11^,^12^,^64^. I focus on four early crown mammal groups: eutricondontans, spalacotherioids, dryolestoids, and therians (and close kin) (Fig. 1). Multituberculates are excluded because of their palinal (rather than orthal and transverse) jaw movement during occlusion. Average results of the morphometric analyses for each group (Figs 3 and 4) are used as the framework for determining jaw joint elevation, relative elevation of the coronoid process as an insertion point for the temporalis muscle (TM), and muscle insertion points for the superficial masseter (SM) and medial pterygoid (MP) muscles.

Since very few 3D skulls of Mesozoic mammals have been preserved in the fossil record, locations of muscle origins (for determining muscle vectors) were inferred from measurements of 31 extinct mammaliaforms and modern analogs of early mammals (Supplementary Table S2). The SM origin is the approximate location of the anterior zygoma, and the MP origin is the center of the pterygoid process^68^,^83^,^84^. In the models, the TM vector is directed posteriorly and slightly dorsally, which is how it is reconstructed in many extant mammals^84^ and stem mammaliaforms^3^,^5^,^9^. Muscle origin locations are kept constant for the results in Fig. 6. However, variation associated with potential changes to these locations among mammal groups was examined, and results are reported in the Supplementary information. Jaws are assigned an *x* axis length of 10 distance units (d.u.) and a maximum *y* axis width of 6 d.u. (at the jaw joints), with this length-width ratio based on measurements of modern and fossil mammals (Supplementary Table S2).

To determine force contributions of each muscle to a particular type of rotation, the line representing the muscle (i.e., the line drawn between the muscle origin and insertion in 3D) is first separated into *x, y*, and *z* components (see Fig. 2 for coordinate planes). Each component (e.g., *y* in Equation 1) is then divided by the muscle length (*d*) and multiplied by the assigned force (*F*) of that muscle, resulting in the force contribution of the muscle in a single direction. For example, the force contribution (*F*_y_) of the *y* axis component (*y*) of one muscle is:


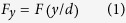


Force assignments (*F*) in Equation 1 are relative muscle masses (i.e., each value is a percentage of the total mass of the jaw musculature). The relative sizes and forces of jaw muscles are expected to vary considerably among mammals, and therefore multiple force assignments are used. These values are based on relative muscle masses reported by Turnbull^84^ for *Didelphis virginiana* (TM, 0.57; SM, 0.14; and MP, 0.07), *Echinosorex gymnurus* (TM, 0.61; SM, 0.11; and MP, 0.09), and *Canis familiaris* (TM, 0.67; SM, 0.10; and MP, 0.03). These species were chosen because they represent distinct mammalian orders and possess unique diets: *D. virginiana*, generalist; *E. gymnurus*, insectivore; and *C. familiaris*, carnivore. The deep masseter is excluded from analyses because it is not one of the Triplet muscles (as defined by Weijs^56^) and therefore is not expected to reach peak contraction concurrently with Triplet muscle groups, making it difficult (or unnecessary) to include in the models. Further, the deep masseter does not insert on the jaw processes (i.e., AP and coronoid process) that are a large focus of this study, making it difficult to track the evolutionary changes to this muscle. See the Supplementary information for additional discussion.

Moment arm (i.e., in-lever) lengths (*L*) are the lengths of the perpendicular lines from the *x, y*, and *z* vectors to the axis of rotation (see Fig. 5 for an example in 2D). (For simplicity, these lengths are treated as positive values in calculations unless the force vector works against the direction of rotation). To calculate the moment (*M*) about an axis of rotation, the moment arm lengths are multiplied by the relevant *x, y*, and *z* force components (e.g., results of Equation 1 for the *y* component), and results are summed. Finally, the moments for the working-side SM, working-side MP, and balancing-side TM (i.e., Triplet II muscle group) are summed to produce the total moment for the mandible. As an example, the total moment for pitch rotation around a mediolateral oriented axis (in which *F*_z_ would not contribute because it is parallel to the axis of rotation) is calculated with the equations:


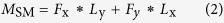



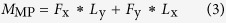



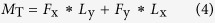



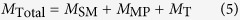


The resulting moments are nearly proportional to the mechanical advantages of the muscle vectors since the distance from the bite point to the axis of rotation (i.e., out-lever) is not treated as a variable and is expected to remain very similar among groups (see the Supplementary information). Calculations are repeated for average musculoskeletal configurations of eutricondontans, spalacotherioids, dryolestoids, and therians.

Moments are calculated for a mediolaterally oriented axis (i.e., *z* axis) of rotation through the jaw joints for pitch rotation, an oblique axis through the jaw joint and mandibular symphysis for roll rotation, and two dorsoventrally oriented axes (i.e., *y* axes) of rotation for yaw rotation (Fig. 6). For roll rotation, only a single hemimandible is used in the models because the mandibular symphysis of early mammals is likely unfused and permits independent rotation of hemimandibles^6^,^50^. For yaw rotation, two dorsoventrally oriented axes are used because the location of this axis is likely variable both among mammals and during chewing cycles in individuals^46^,^48^,^49^,^52^,^53^,^54^,^55^. One is placed just medial to the balancing (i.e., non-chewing) side jaw joint. A second axis is placed on the midline (i.e., sagittal plane) of the jaw and at 75% of the jaw length, similar to the position predicted for some primates^55^.

See the Supplementary information for extended methodology.

## Additional Information

**How to cite this article**: Grossnickle, D. M. The evolutionary origin of jaw yaw in mammals. *Sci. Rep.* **7**, 45094; doi: 10.1038/srep45094 (2017).

**Publisher's note:** Springer Nature remains neutral with regard to jurisdictional claims in published maps and institutional affiliations.

## Supplementary Material

Supplementary Information
