# Supplementary material for: The evolutionary origin of jaw yaw in mammals
Source: Sci Rep. 2017 Mar 21;7:45094. doi: 10.1038/srep45094 (PMC5359619; doi:10.1038/srep45094)
Supplement: Supplementary Information [file srep45094-s1.pdf]

Supplementary Information for

The evolutionary origin of jaw yaw in mammals

David M. Grossnickle

Committee on Evolutionary Biology, University of Chicago, IL

Contents

Part A. Mammal Groups

Part B. Extended Methods

Supplementary Figure S1. PCA using semilandmarks and sliding semilandmarks

Supplementary Figure S2. Thin plate spline using semilandmarks and sliding semilandmarks

Supplementary Table S1. Jaw specimens and ages

Supplementary Figure S3. Jaw models with labeled 3D coordinates

Part C. Extended Results and Discussion

Supplementary Table S2. Morphometrics: jaw joint, coronoid process, and tooth row

Supplementary Table S3. Morphometrics: jaw dimensions and muscle origins

Supplementary Figure S4. Angular process morphospace plots with taxa labels

Supplementary Table S4. Moment values

Supplementary Figure S5. Sensitivity test for variation in muscle origin locations

Part D. Supplementary Information References

PART A. MAMMAL GROUPS

Prior to morphometric analyses, stem mammaliaform and early crown mammal genera were assigned to major groups. These groups are based primarily on the strict consensus phylogenies of Martin *et al.* (2015), Luo *et al.* (2015A), and Luo *et al.* (2015B), which all use derivatives of a similar phylogenetic character matrix. The phylogenetic relationships of early mammal groups is important for inferences made in this study, and the following section includes discussion of issues related to these groupings.

It is worth noting that a benefit of considering the average jaw shapes of major groups is that it helps minimize the error associated with individual jaws and potential incorrect phylogenetic assignments of genera. For instance, many jaw images are reconstructions or photographs of incomplete jaws. Thus, the accuracy of specific jaw morphologies may be questionable in some cases. However, by considering the average jaw morphology for each major mammal group, the effect of this error on broad evolutionary trends is minimized.

Stem mammaliaforms. The stem mammaliaforms group is paraphyletic and includes *Sinoconodon*, morganucodontids, *Hadrocodium*, docodonts and haramiyids. The phylogenetic position of haramiyids is contentious. For instance, I follow Martin *et al.* (2015) and Luo *et al.* (2015A) and treat haramiyids as stem mammaliaforms, but haramiyids have also been hypothesized to be crown mammals that are sister to multituberculates (e.g., Bi *et al.* 2014). Examining how alternate phylogenetic positions of this group would affect results is beyond the scope of this study. However, it is unlikely that moving haramiyids would have a major effect on results. For instance, stem mammaliaforms (including haramiyids) and multituberculates both occupy the right side of the angular process (AP) geometric morphometric morphospace (PC1 and PC2) in Figure 3. Thus, moving haramiyids to the multituberculate group (*sensu* Bi *et al.* 2014) would not affect the broad conclusions concerning the evolution of the AP. Also, neither stem mammaliaforms nor multituberculates are included in the jaw model analyses, so the placement of haramiyids does not affect these analyses.

Australosphenidans. Australosphenidans are commonly recovered in cladistic analyses as stem monotremes that branch from the crown mammalian node (Bi *et al.* 2014, Krause *et al.* 2014, O'Meara and Thompson 2014, Martin *et al.* 2015, Luo *et al.* 2015A, Luo *et al.* 2015B). In these analyses, shuotheriids (which include *Pseudotribos* of this study) are generally recovered as the sister group to australosphenidans, forming a monophyletic clade. Thus, I include *Pseudotribos* with the australosphenidans in this study.

It has been hypothesized that australosphenidans are more closely related to (or members of) Theria rather than stem monotremes (Rich *et al.* 1997, Rich *et al.* 2002). However, it is unlikely that altering the phylogenetic position of australosphenidans would have a large impact on the broad patterns observed in this study. For instance, some austalosphenidan jaws (e.g., *Henosferus*) are morphologically very similar to therian jaws (Figs. 3-4), meaning that if australosphenidans are placed with the therian group (*sensu* Rich *et al.* 1997) it is not expected to have a major effect on the morphometric and modeling results for therians.

Multituberculates. Multituberculates included in this study are limited to plagiaulacidan multituberculates because preserved jaws of cimolodontan multituberculates do not appear in the fossil record until the Late Cretaceous, which is after the time period of interest. Despite being closely related to additional crown mammal groups of this study (Fig. 1), multituberculates are excluded from the jaw modeling analyses because of their palinal (rather than orthal and transverse) jaw movement. See Gambaryan and Kielan-Jaworowska (1995) and Wall and Krause (1992) for considerations of multituberculate feeding mechanics.

Eutriconodontans. Phylogenetic analyses often recover Eutriconodonta as a monophyletic clade of early crown mammals that are outside of Cladotheria (Bi *et al.* 2014, Krause 2014, Martin *et al.* 2015, Luo *et al.* 2015A), although some analyses recover the group as stem mammals (Rougier *et al.* 2011, Rougier *et al.* 2012, O'Meara and Thompson 2014). Due to the early-branching position of eutriconodontans and the primitive triconodont dentition, it could be argued that eutriconodontans possess primitive jaw and molar morphologies that are ancestral to spalacotherioids and cladotherians. This is supported by the similarities of the posterior jaw processes between eutriconodontans and multituberculates (Figs. 3 and 4), which are also early-branching crown mammals. However, it is worth noting that the eutriconodontan taxa may possess derived morphologies that are not representative of the ancestral condition. For instance, many eutriconodontans (e.g., *Repenomamus*) are relatively large and carnivorous (Hu *et al.* 2005B), whereas most spalacotherioids and early cladotherians are small and likely insectivorous (Grossnickle and Polly 2013, Kielan-Jaworowska *et al.* 2004). Thus, eutriconodontans may have jaw and molar morphologies that are adapted for a derived dietary preference. Even if this is the case, however, eutriconodontan jaws represent a functional morphology that is significantly different than those of cladotherians. Thus, it still provides an opportunity for comparative analyses of different functional morphologies among closely related early mammal groups.

Spalacotherioids. Spalacotherioids are acute-angled "symmetrodonts" that are members of Trechnotheria (Kielan-Jaworowska *et al.* 2004, Martin *et al.* 2015).

The phylogenetic position of tinodontids varies among cladistic analyses (Bi *et al.* 2014, Krause *et al.* 2014, O'Meara and Thompson 2014, Martin *et al.* 2015, Luo *et al.* 2015A), and *Tinodon* is recovered in Martin *et al.* (2015) in a polytomy with eutriconodontans, multituberculates, and trechnotherians (i.e., spalacotherioids and cladotherians). However, they possess symmetrodont molars and are often grouped with (or recovered in phylogenetic analyses near) symmetrodont taxa such as spalacotherioids (e.g., Kielan-Jaworowska *et al.* 2004, Luo *et al.* 2015, Martin *et al.* 2015). Thus, I tentatively place the two tinodontids of this study (i.e., *Tinodon* and *Yermakia*) with spalacotherioids. *Yermakia*'s angular process (AP) region is not preserved (Lopatin *et al.* 2005), and therefore only *Tinodon* is included in the geometric morphometrics (GM) analysis of AP shape. Consistent with both eutriconodontans and spalacotherioids, a distinct AP is not present in *Tinodon*. Thus, if *Tinodon* were moved to the eutriconodontan group in morphometric analyses of this study, it is expected to have little effect on the results for either clade.

Dryolestoids. This group is a lineage of early stem cladotherians (i.e., "eupantotherians") that possess a eupantothere molar morphology (Figs. 1 and 5). The diagnostic feature of this morphology is a talonid shelf on the lower molars. The dryolestoids examined in this study are

believed to be monophyletic (e.g., Rougier *et al.* 2011, Martin *et al.* 2015). Closely related to dryolestids are meridiolestidans, which are nested within Dryolestoidea in the analysis of Rougier *et al.* (2011). In the phylogenetic analyses of Rougier *et al.* (2012) and O'Meara and Thompson (2014), dryolestids and meridiolestidans (and close kin) form a paraphyletic clade. However, no meridiolestidans are included in this study since they do not appear in the fossil record until the Late Cretaceous, which is after the time period of interest. Further, their closest kin (e.g., *Paurodon*) are not represented in the fossil record by jaws with preserved posterior processes. It is worth noting that the temporal range of dryolestoids in Figure 1 is based on dryolestids and meridiolestidans (Rougier *et al.* 2011).

Therians and close kin. This group includes late-branching stem cladotherians (i.e., *Amphitherium* and peramurans) and early tribosphenidans (i.e. boreosphenidans), which are comprised primarily of therians. The two peramurans in this study are *Peramus* and *Tendagurutherium*. In cladistic analyses, *Amphitherium* and *Peramus* are recovered as sister taxa that form a branch between dryolestoids and Theria (Rougier *et al.* 2012, Bi *et al.* 2014, Krause *et al.* 2014, O'Meara and Thompson 2014, Martin *et al.* 2015, Luo *et al.* 2015A). Peramurans and tribosphenidans form Zatheria (McKenna 1975, Kielan-Jaworowska *et al.* 2004), and therefore the 'therians and close kin' group could also be titled Zatheria + *Amphitherium*.

Amphitherium possesses a lower molar with a talonid that has one cusp, which is likely the hypoconid or hypoconulid (Kielan-Jaworowska *et al.* 2004, Davis 2011), and peramurans possess a talonid with an incipient basin with diminutive cusps (Kielan-Jaworowska *et al.* 2004). In this study, I hypothesize that the talonid shelf and talonid basin have separate functional effects on jaw morphologies and movement during occlusion. Since *Amphitherium* and peramurans have talonids that are considered more derived than the talonid shelves (or hypoflexids) of dryolestoids, it can be argued that their jaws and molars will function more similarly to early therians (which possess talonid basins) than dryolestoids. Thus, I include these taxa with therians rather than with the additional stem cladotherian taxa of this study, dryolestoids. Further, these group assignments form two monophyletic clades (i.e., dryolestoids and Zatheria + *Amphitherium*), but placing *Amphitherium* and peramurans with dryolestoids would result in a paraphyletic stem cladotherian group.

The tribosphenic molar morphology it considered an apomorphy of Tribosphenida (i.e., Boreosphenida) (Luo *et al.* 2002, Kielan-Jaworowska *et al.* 2004). However, no stem tribosphenidans are included in this study, and taxa are often given the taxonomic assignment of stem tribosphenidan when their affinity to a therian group (i.e., Eutheria or Metatheria) is unknown (Kielan-Jaworowska *et al.* 2004). Thus, for simplicity, I use 'therians' instead of 'tribosphenidans' in this study when referring to cladotherian taxa with tribosphenic molars.

Many metatherians possess a medially inflected angular process (AP), which is problematic for 2D morphometric analyses. However, *Sinodelphys* is the only preserved metatherian jaw in the time period of interest and its AP is not inflected, at least as it is preserved (Luo *et al.* 2003).

***Fruitafossor* and *Vincelestes*.** These taxa were included in the morphometric analyses (see Extended Results) but are not included in a specific mammal group due to uncertain phylogenetic affinities and derived features. *Fruitafossor* is recovered as an early crown mammal, but it is a

single-branch lineage that is not nested within any group of this study (Martin *et al.* 2015, Luo *et al.* 2015A, Luo *et al.* 2015B). *Vincelestes* is a stem cladotherian that is also recovered as a single-branch lineage that is not nested within any group of this study. In some phylogenetic analyses it is recovered outside of Zatheria (Theria + peramurans) (Krause *et al.* 2104, Martin *et al.* 2015, Luo *et al.* 2015A, Luo *et al.* 2015B), but in additional analyses it is recovered within Zatheria (Rougier 2012, O'Meara and Thompson 2014).

An additional issue with *Fruitafossor* and *Vincelestes* is that they appear to have derived dental and jaw features that may be due to adaptations for specialist diets. *Fruitafossor* has tubular molars that are indicative of obligate insectivory (Luo and Wible 2005). The jaw of *Fruitafossor* is unique in possessing a distinct (yet diminutive) and inflected AP, and a small coronoid process that is at the approximate elevation of the raised jaw joint. This combination of dental and jaw features is not present in any mammal group of this study. In addition, *Vincelestes* demonstrates characters indicative of carnivory, such as very large canines and a short tooth row (Rougier 1993). Like *Fruitafossor*, it possesses jaw traits that are unique to any other group in this study. The coronoid process is extremely elevated relative to the molar row, which is common for many modern and extinct carnivorous mammals (including eutriconodontans such as *Repenomamus*). However, unlike most carnivores, it also has a relatively elevated jaw joint and, unlike eutriconodontans, it has a distinct AP. The tooth row relative to the length of the jaw is also shorter than crown mammals of this study besides several multituberculates. Thus, if *Fruitafossor* and *Vincelestes* were included with any group of this study they would be outliers in morphometric analyses, and they are unlikely to be ideal representatives of morphologies of any group.

PART B. EXTENDED METHODS

Specimens. Images of stem mammaliaform and early crown mammal jaws were collected from the primary literature. Sources, geologic ages, and specimen information are provided in Supplementary Table S1. If more than one jaw specimen is known for a genus, the best-preserved (or most complete) specimen was chosen to represent the genus. All specimens are from the latest Triassic through Early Cretaceous (i.e., ~210-100.5 Ma). This time period was chosen because it captures all phylogenetic nodes of interest for this study and includes a substantial sample of taxa. In addition, only two major groups of mammals (i.e., cimolodontan multituberculates and crown therians) remain diverse after the mid-Cretaceous (Grossnickle and Polly 2013), at least in Laurasian landmasses, and both groups experience an ecomorphological radiation in the Late Cretaceous in which evolutionary changes in jaw shape are likely associated with increased dietary diversity (Wilson *et al.* 2012, Grossnickle and Polly 2013, Grossnickle and Newham 2016). Thus, I believe that truncating the study at the mid-Cretaceous is important for examining the primitive morphologies of therians and multituberculates.

Supplementary Table S1. Specimen information for the mammaliaform genera analyzed in this study. Additional notes concerning the specimens are provided in Supplementary Table S3. Abbreviations: Cret., Cretaceous; E., Early; Jur., Jurassic; L., Late; M., Middle; Tri., Triassic.

Taxon	Image source	Specimen	Age
**Stem Mammaliaforms**			
Agilodocodon	Meng *et al.* 2015	Reconstruction based on BMNH001138A and B	M. Jurassic
Arboroharamiya	Zheng *et al.* 2013	Reconstruction based on STM33-9	M. or L. Jur.
Castorocauda	Ji *et al.* 2006	Reconstruction based on JZMP 04-117	M. Jurassic
Docodon	Simpson 1929	Reconstruction based primarily on YPM 11826.	L. Jurassic
Docofossor	Luo et a. 2015B	Composite reconstruction using micro-CT scans of BMNH131735A and B	L. Jurassic
Dinnetherium	Jenkins *et al.* 1983	MCZ 20872	E. Jurassic
Hadrocodium	Luo *et al.* 2001	IVPP 8275	E. Jurassic
Haldanodon	Luo 2011	Composite reconstruction based on multiple specimens, including those in Lillegraven & Krusat (1991).	L. Jurassic
Haramiyavia	Luo *et al.* 2015A	Composite reconstruction using micro-CT scans of MCZ7/95A and B.	L. Triassic
Kuehneotherium	Gill *et al.* 2014	Composite reconstruction based on micro-CT scans of multiple specimens.	L. Triassic-E. Jurassic
Megazostrodon	Kielan-Jaworowska *et al.* 2004	Composite drawing based on multiple specimens.	E. Jurassic
Morganucodon	Gill *et al.* 2014	Composite reconstruction based on micro-CT scans of multiple specimens.	L. Triassic-E. Jurassic
Sinoconodon	Zhang *et al.* 1998	IVPP 4727	L. Tri.-E. Jur.
Megaconus	Zhou *et al.* 2013	Reconstruction based on PMOL-AM00007A and B	M. Jurassic
Xianshou	Bi *et al.* 2014	Reconstruction of IVPP V16707A	L. Jurassic
**Australosphenidans**			
Asfaltomylos	Rauhut *et al.* 2002	MPEF-PV1671	E. Jurassic
Bishops	Rich *et al.* 2001		E. Cretaceous
Henosferus	Rougier *et al.* 2007	Composite reconstruction based on multiple specimens.	E. Jurassic
Pseudotribos	Luo *et al.* 2007B	Reconstruction based on CAGS040811	M. Jurassic
Teinolophos	Rich *et al.* 2016	MSC 148 (NMV P20823)	E. Cretaceous
**Multituberculates**			
Ctenacodon	Simpson 1929	Reconstruction based on YPM 11833.	L. Jur.-E. Cret.
Guimarotodon	Hahn & Hahn 1998	Reconstruction based on Nach V.J. 461-155 and IPFUB Gui Mam 143/76	L. Jurassic
Kuehneodon	Hahn 1969	Reconstruction based on V. J. 4-155	L. Jurassic
Meketibolodon	Hahn & Hahn 1998	IPFUB Gui Mam 89/76	L. Jurassic
Paulchoffatia	Hahn 1969	Reconstruction based on V. J. 1-155	L. Jurassic
Plagiaulax	Simpson 1929	Composite reconstruction based on multiple specimens.	M. Jurassic
Rugosodon	Yuan *et al.* 2013	Composite reconstruction based on BMNH1143A and B	L. Jurassic
Sinobaatar	Hu & Wang 2002	IVPP V12517	E. Cretaceous
Zofiabaatar	Bakker & Carpenter 1990		L. Jurassic
**Eutriconodontans**			
Amphilestes	Simpson 1928	Composite reconstruction based on multiple specimens.	M. Jurassic
Argentoconodon	Gaetano & Rougier 2011	Reconstruction based largely on MPEF-PV2363	E. Jurassic
Gobiconodon	Jenkins & Schaff 1988	MCZ 19965	E. Cretaceous
Jeholodens	Ji *et al.* 1999	Drawing of GMV 2139	E. Cretaceous
Liaoconodon	Meng *et al.* 2011	Reconstruction based on IVPP V16051	E. Cretaceous
Phascolotherium	Simpson 1928	Composite reconstruction based on multiple specimens.	M. Jurassic
Priacodon	Kielan-Jaworowska *et al.* 2004	Composite reconstruction based on multiple specimens.	L. Jurassic
Repenomamus	Wang *et al.* 2001	IVPP V12549	E. Cretaceous

Triconodon	Simpson 1928	Composite reconstruction based on multiple specimens.	E. Cretaceous
Trioracodon	Simpson 1928	Composite reconstruction based on multiple specimens.	L. Jur.-E. Cret.
Volaticotherium	Meng *et al.* 2006	Reconstruction based on IVPP V14739	M. Jurassic
Yanoconodon	Luo *et al.* 2007A	Reconstruction based on NJU-P06001A	E. Cretaceous
**Spalacotherioids (& *Tinodontids*)**			
Heishanlestes	Hu *et al.* 2005A	IVPP V 7480	E. Cretaceous
Maotherium	Ji *et al.* 2009	Reconstruction based on HGM 41H-III-0321	E. Cretaceous
Spalacotherium	Kielan-Jaworowska *et al.* 2004		E. Cretaceous
Tinodon	Simpson 1925	Composite reconstruction based on multiple specimens.	L. Jur.-E. Cret.
Yermakia	Lopatin *et al.* 2005	PM TGU 16/7-22	E. Cretaceous
Zhangheotherium	Hu *et al.* 1997	Reconstruction based on IVPP V7466	E. Cretaceous
Lactodens	Han & Meng 2016	HG-M016	E. Cretaceous
**Dryolestoids**			
Amblotherium	Simpson 1928	Composite reconstruction based on multiple specimens.	L. Jur.-E. Cret.
Crusafontia	Krebs 1993	Composite reconstruction based on multiple specimens.	E. Cretaceous
Dryolestes	Martin 1999	Gui Mam 41/79	L. Jurassic
Henkelotherium	Luo 2007	Composite reconstruction based on multiple specimens, including those in Henkel & Krebs (1977) and Krebs (1991)	L. Jurassic
Laolestes	Prothero 1981		L. Jur.-E. Cret.
Phascolestes	Simpson 1928	Composite reconstruction based on multiple specimens	E. Cretaceous
**Therians & close kin**			
Eomaia	Ji *et al.* 2002	Reconstruction based on CAGS01-IG1-a and b	E. Cretaceous
Juramaia	Luo *et al.* 2011	Reconstruction based on BMNH PM1143	M. or L. Jur.
Montanalestes	Cifelli 1999	Reconstruction based on OMNH 60793	E. Cretaceous
Peramus	Prothero 1981		E. Cretaceous
Prokennalestes	Kielan-Jaworowska & Dashzeveg 1989	GI PST 10-6 (for AP analysis), GI PST 10-14b (for additional measurements)	E. Cretaceous
Sasayamamylos	Kusuhashi *et al.* 2013	MNHAH D1-030444	E. Cretaceous
Sinodelphys	Luo *et al.* 2003	Reconstruction based on CAGS00-IG03	E. Cretaceous
Tendagurutherium	Heinrich 1998	MB.Ma.46910	L. Jurassic
Amphitherium	Simpson 1928		M. Jurassic
**Other**			
Vincelestes	Bonaparte & Rougier 1987		E. Cretaceous
Fruitafossor	Luo & Wible 2005	Reconstruction based on LACM 150948	L. Jurassic

Jaw measurements. The linear jaw measurements and location of geometric morphometrics (GM) landmarks of this study are briefly explained in the main text. In addition, the region of landmarks for the angular process (AP) shape analysis is illustrated in Figure 3, and elevation measurements for the coronoid process and jaw joint (i.e., condylar process) are shown in Figure 4. However, additional details are provided here.

To measure jaw joint elevation (or depression), an extended line was first drawn from the alveolar margin (i.e., dorsal edge of the jaw body at the base of the molar row) posteriorly to the condylar process. (I chose to use this line rather than a line from the molar cusps because molars are not always preserved in fossil jaws. Further, molar cusps are often worn or include multiple occlusal surfaces at different elevations (e.g., tribosphenic lower molars include elevated shearing crests on

the trigonid and wear surfaces on the depressed talonid basin), making it especially difficult to determine a horizontal line based on the molars.) From the initial line, a perpendicular line was drawn to the jaw articulation surface (i.e., jaw joint), or posterodorsal-most point of the condylar process. I used the midpoint of the articulation surface for genera with an extensive articulation surface (e.g., multituberculates). The length of this line was measured as the jaw joint elevation (or depression), and dividing this value by jaw length standardized the measurements.

I measured the length of the tooth row as a means of testing whether the typical out-lever length (i.e., distance from the axis of rotation to the bite point) was likely to vary significantly among mammal groups. My assumption is that similar tooth row lengths relative to jaw length will result in similar bite point locations. Conversely, if one mammal group has significantly longer tooth rows in which molars are more posteriorly positioned, I would expect that the typical bite points during molar occlusion are also relatively posterior in position.

The tooth row length was measured from the base of the anterior-most incisor to the posterior edge of the ultimate molar, and this was then standardized by dividing by the length of the jaw. For a few genera, the anterior tips of jaws are not preserved, and in these cases the jaw and tooth row lengths were estimated. Results for this measurement are not shown in the main text but are provided in the Extended Results of the Supplementary Information.

Geometric morphometrics (GM). For the GM analysis of the AP region of the jaw (Fig. 3), one landmark was placed between the ultimate and penultimate molars. This landmark served to maintain the correct polarity for the outline during the Procrustes analysis (otherwise, the Procrustes analysis might flip outlines vertically to better align morphologies), and capture variance associated with thickness of the jaw body and elevation/depression of the AP. The semilandmarks begin along the ventral edge of the jaw, at a spot that is perpendicular to the base of the molar row (i.e., the horizontal line at the alveolar margin that was also used in the linear morphometric measurements). See the discussion below for reasons why semilandmarks were used instead of sliding semilandmarks. The landmarks end at the ventral-most point of the articulation surface (or head of the condylar process).

Ideally, landmarks and semilandmarks of GM analyses should represent homologous points of a structure, but this is unlikely to be the case for many landmarks/semilandmarks in this study. For example, the number of molars varies among mammal groups, so the single landmark between the ultimate and penultimate molars is unlikely to be homologous among all taxa. In addition, the presence/absence of the AP varies among mammal groups, meaning that the semilandmarks cannot possibly capture homologous points if some taxa have structures that other taxa lack. However, I consider these landmarks to still fall within the ‘Type III’ landmark category of Bookstein (1997) because they are outlining the same region of the jaw in all taxa and likely capturing homologous muscle insertion locations for the SM and MP. Also, the goal of the GM analysis of the AP region is simply to quantify the shape of the posteroventral region of the jaw to examine the variation among early mammal groups. In particular, the results help demonstrate that the position of the AP in stem mammaliaforms (i.e., a relatively anterior position) is different than the position of the AP in cladotherians (i.e., a relatively posterior position) (Fig. 3). This can be demonstrated through

qualitative descriptions of the jaws as well, but the GM analysis provides strong support for this observation with quantitative evidence.

The landmark and semilandmark coordinates were subjected to standard geometric morphometrics procedures, which include a Procrustes analysis and a principal components analysis (PCA). The Procrustes analysis realigns shapes (in this case, sets of *x* and *y* coordinates representing shapes) to eliminate variation associated with size (i.e. scaling), translation, and rotation. This results in shapes (represented by Procrustes values) that only vary in terms of shape differences. The Procrustes values are then ordinated using a PCA. Results for the first two principal components (i.e., PC1 and PC2) are shown in Figure 3 (and Supplementary Figure S4 of the Extended Results). In the Figure 3 plots, PC1 is the horizontal axis and accounts for 54.5% of variance, and PC2 is the vertical axis and accounts for 17.4% of variance. The mean shape for the APs was calculated for each mammalian group using all Procrustes values, and the position of these are marked on the PC1 and PC2 morphospace plots of Figure 3 as large, black points. The thin plate splines of the average shapes for groups are shown in middle column of Figure 3. To determine the locations for the SM and MP muscle insertion in the jaw models (Fig. 6), the thin plate splines were overlaid atop one another (see below).

The Procrustes analysis, PCA, calculation of group means, and production of thin plate splines (Fig. 3) were performed using Wolfram's Mathematica and Polly Morphometrics for Mathematica (2016). See the User's Guide for Polly Morphometrics for Mathematica (available at http://mypage.iu.edu/~pdpolly/) and citations within for additional information.

Semilandmarks versus sliding semilandmarks. It is common for geometric morphometric studies that use equally spaced semilandmarks to adjust the semilandmarks by 'sliding' them along tangents of the shape outline to minimize 'bending energy' or Procrustes distances (e.g., Gunz *et al.* 2005, Grossnickle and Newham 2016), helping to increase the likelihood of capturing homologous points along an outline. However, I did not use sliding semilandmarks in this study for a couple reasons. First, the PCA results for analyses using sliding semilandmarks and non-sliding semilandmarks are very similar. Supplementary Figure S1 shows PC1 and PC2 results from my original analysis (left) and results using sliding semilandmarks (right) produced using the Geomorph package (Adams and Otárola-Castillo 2013) for R 3.2.4 (R Core Team 2016). Dryolestoids (red) and therians (orange) are highlighted. Note that dryolestoids are outliers along PC1 (horizontal axis) in both analyses, which is a key result for my conclusions. The morphospace positions of multituberculates and stem mammaliaforms are most affected by using sliding semilandmarks, but those two groups are of less interest in this study.

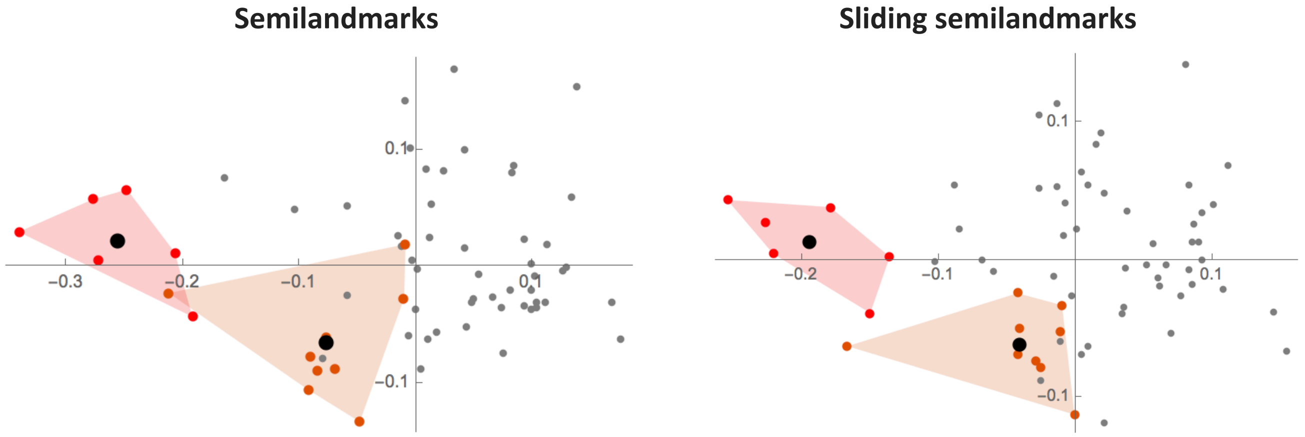

Supplementary Figure S1. Principal component (PC) analysis results for PC1 (horizontal axis) and PC2 (vertical axis) from the original analysis with non-sliding semilandmarks (left; Fig. 3) and a repeated analysis using sliding semilandmarks (right). Dryolestoids are red and therians (and close kin) are orange. Note the similar morphospace positions for the polygons between the two analyses. See Figure 3 for additional details.

Second, sliding the semilandmarks disrupts the original shapes by moving the semilandmarks along tangents of the outline. In the analysis of this study, semilandmarks on the posterior tip of prominent angular processes are 'slid' away from the tip to minimize Procrustes distance (the default method in Geomorph). Gunz *et al.* (2005) note a similar problem with sliding semilandmarks (Figure 8a in Gunz *et al.* 2005) and suggest that this error can be corrected by adding an additional landmark at the tip of the extended region. However, this correction is not possible in my analysis because of a lack of clear homologous points due to many taxa (multituberculates, spalacotherioids, and eutriconodontans) not having an angular process. Thus, the resulting thin plate splines using sliding semilandmarks do not capture the shape of the angular process as well as non-sliding semilandmarks. I demonstrate this in Supplementary Figure S2 with thin plate splines for the mean shape of dryolestoids from the original analysis (left) and from the analysis using sliding semilandmarks (right). This issue is especially problematic because the average shapes produced with sliding semilandmark analyses are less informative (and may not be accurate due to the sliding) for inferring the coordinates used in the jaw models. For example, it is difficult to know from the sliding semilandmark spline how posteriorly extended the angular process is, and this posterior point is important for estimating the muscle insertion locations for the medial pterygoid and superficial masseter. Also, the average angular process shape would simply not be illustrated as clearly in Figure 3 for the reader if sliding semilandmarks were used (Supplementary Figure S2).

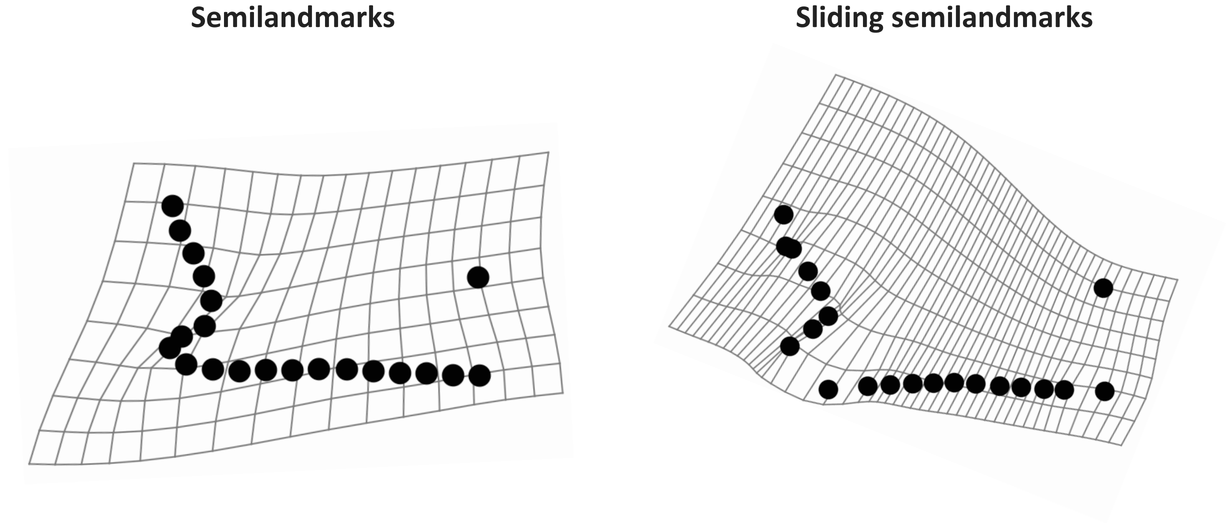

Supplementary Figure S2. Thin plate splines for the mean shape of dryolestoid angular processes from the original analysis with non-sliding semilandmarks (left; Fig. 3) and from a repeated analysis using sliding semilandmarks (right). See Figure 3 for additional details.

Norman MacLeod explains these two problems (and an additional issue) with sliding semilandmarks in more detail and provides further examples: http://cdn.palass.org/palaeomath_101/moribund/downloads/Landmarks_Semilandmarks.pdf. The 'relaxation step' described by Gunz *et al.* (2005) may help alleviate the second issue by returning the semilandmark from the tangent line to the original outline, but I do not think this would help analyses of this study because the similar problem that they address (Figure 8a from Gunz *et al.* 2005) still occurred even with the relaxation step.

Two-dimensional analyses. One potential concern with the morphometrics analyses of this study is that the methods only capture two dimensions of the jaw. This concern is highlighted by the fact that marsupials (i.e., metatherians) tend to possess a prominent, medially inflected AP that cannot be well represented by two-dimensional (2D) landmarks. However, 2D analyses of jaw function allow for a much greater sample size and are commonplace in the literature. Therefore, it is expected that broad evolutionary patterns and functional considerations can be obtained from the 2D jaw morphometric analyses performed in this study. Further, only one jaw of a metatherian, *Sinodelphys*, is preserved from the time period of this study, and it does not possess an inflected AP (Luo *et al.* 2003).

Jaw models – measurements. To examine functional changes in therians and their close ancestors, 3D jaw models were constructed for eutriconodontans, spalacotherioids, dryolestoids, and early therians (and close kin) (Figs. 5-6). These were produced using Wolfram's Mathematica. See Figure 2 for the x, y, and z coordinate system used for the models.

In the jaw models, the base of the tooth row (i.e., dorsal edge of the jaw body, or alveolar margin) is set as a horizontal line at $y = 0$. The y-axis length from the mandibular symphysis to the

jaw joint is 10 distance units (d.u.), and this is kept constant for all jaw models. For each mammal group, the average (median) jaw joint elevation above (or depression below) the base of the molar row (Fig. 4) is used to assign the vertical (i.e., y-axis) jaw joint locations (Fig. 6, Supplementary Fig. S3). For instance, the average jaw joint elevation for dryolestoids is 12.6% of the jaw length. Thus, the *y*-axis value for the dryolestoid jaw joint in the model is 12.6 d.u. Similarly, the average (median) coronoid process elevations (Fig. 4) were used for each group and represent the approximate muscle insertion locations for the TM. (It is recognized that the TM includes an extended attachment along the dorsal edge of the coronoid process, and the y-axis location for the center of the force vector could vary among groups. However, examining these variables is beyond the scope of this study.) See the Extended Results (Supplementary Table S3) for additional median values of jaw joint elevation and coronoid process elevation that were used in the models.

To determine the *x* and *y* coordinates for the SM and MP muscle insertion locations, thin plate splines from the GM analysis of AP shape (Fig. 3) were overlaid atop one another with the single landmark (which is between the ultimate and penultimate molars) aligned. The splines were horizontally stretched so that the length between the single landmark and the posterior-most semilandmark is equal for all splines. For the dryolestoid and therian groups, the SM and MP insertion location is assigned to the posterior-most point along the edge of AP. For spalacotherioids and eutriconodontans, which don't have a distinct AP, considering the location of the posterior portion of the masseteric fossa assisted in determining the central location of the insertion site.

It is worth noting that although the AP is relatively gracile in many early cladotherians, this does not necessarily indicate that only a small amount of muscle attaches to the AP. For instance, the MP and SM of modern mammals tend to produce a large, muscular sling that wraps around the AP and the posteroventral region of the jaw (Turnbull 1970). Even taxa such as shrews that have an elongate and very thin AP maintain a considerable amount of muscle mass in the AP region.

To determine jaw dimensions (i.e., length and posterior width) to use for the models, direct measurements of specimens could not be used for early mammal genera for a couple reasons. First, preserved fossils of intact lower jaws with both hemimandibles are extremely rare. Further, it is unlikely that those that are preserved maintain a reliable angle of attachment at the mandibular symphysis since many fossils are flattened or distorted. Second, even if both hemimandibles are preserved intact, the angle at the symphysis remains unreliable because early mammals are believed to have had unfused symphyses, which allows for 'wishboning' of the jaw and changes to the angle at the symphysis.

Thus, jaw length and width for the models was determined using the average dimensions of six extinct mammaliaforms (including three crown mammals) and 25 extant mammal genera (Supplementary Table S2). The extant mammals chosen for measurements are primarily small insectivores or omnivores that are appropriate analogs of Mesozoic mammals. In addition, they are taxonomically diverse, representing several orders of eutherians and metatherians. Measurements of extant mammals were taken at the Field Museum of Natural History (FMNH), and measurements of extinct mammals were obtained from images in the published literature (sources given in Supplementary Table S2). Since many mammalian species have unfused mandibular symphyses, museum preparators often glue hemimandibles together, meaning that the angle at the symphysis

(and width of the jaw) is not reliable for many specimens. Thus, jaw width measurements were taken as the distance from the centers of the articular surfaces of the glenoid fossae of the skull. These measurements were then divided by the jaw length. Results indicate that the average jaw width at the jaw joint (or glenoid fossae) is approximately 60% of jaw length. Hence, the jaw width for the models was set at 6 d.u. and the length was set at 10 d.u. These values were kept constant for eutriconodontans, spalacotherioids, dryolestoids, and early therians (and close kin).

Supplementary Table S2. Measurements of mammaliaform and modern mammal jaws and skulls. Average jaw measurements are used for determining the length and posterior width of the jaw models (Fig. 6, Supplementary Fig. S3). Average skull measurements are used to determine the estimated position of the muscle origins of the superficial masseter (SM) and medial pterygoid (MP) (see text). Posterior jaw widths at the jaw joints are often estimated from the glenoid fossae of the skull (see text). 'Pterygoid to pterygoid width' and 'zygoma to zygoma width' values are divided by the total jaw width at the jaw joints. Length measurements (i.e., 'anterior tip of jaw to pterygoid' and 'anterior tip of jaw to zygoma') are divided by the total jaw length. Thus, table values represent ratios of the measurements to the total width or length of the jaw. Values in bold and italics are averages (means) for each group, and overall means, medians, and standard deviations are given at the bottom. Field Museum of Natural History (FMNH) specimen numbers are given in parentheses after the genus name.

	**MP origin (i.e., pterygoid process) location**		**SM origin (i.e., anterior zygoma) location**		**Jaw dimenstions**
**Taxon**	**Pterygoid to pterygoid width**	**Anterior tip of jaw to pterygoid**	**Zygoma to zygoma width**	**Anterior tip of jaw to zygoma**	**Jaw width to jaw length**
**Fossil mammaliaforms**	***0.2476***	***0.7417***	***0.8631***	***0.5383***	***0.6540***
Morganucodon (Kielan-Jaworowska *et al.* 2004)	0.3400	0.6235	0.7120	0.5134	0.6181
Sinoconodon (Zhang *et al.* 1998)	0.2733	0.7080	0.8109	0.5204	0.6551
Hadrocodium (Luo et al. 2001)	0.2887	0.7025	0.8017	0.5218	0.6741
Vincelestes (Rougier 1993)	0.2108	0.7525	0.8476	0.4495	0.6950
Mayulestes (de Muizon 1998)	0.1674	0.8616	1.0023	0.5700	0.6333
Asiatherium (Kielan-Jaworowska *et al.* 2004)	0.2053	0.8024	1.0041	0.6548	0.6482
**Modern eutherians**	***0.2446***	***0.8459***	***0.9966***	***0.5688***	***0.6002***
Amblysomus (FMNH 165582)	0.1891	0.7703	0.7264	0.4363	0.8056
Chrysochloris (FMNH 207280)	0.2356	0.7648	0.7737	0.5020	0.8698
Echinosorex (FMNH 85105)	0.2451	0.8288	1.0602	0.6081	0.4253
Erinaceus (FMNH 96345)	0.4140	0.8503	1.0740	0.5292	0.7125
Hylomys (FMNH 32308)	0.3683	0.8642	0.8824	0.5836	0.6191
Solenodon (FMNH 57320)	0.1527	0.8375	1.1782	0.5745	0.4558
Scutisorex (FMNH 160178)	0.1724	0.8844	0.9009	0.5260	0.6831
Suncus (FMNH 57608)	0.1005	0.8999	0.9308	0.4688	0.6374
Talpa (FMNH 6449)	0.2006	0.8216	0.8382	0.5969	0.5377
Condylura (FMNH 199405)	0.3329	0.8683	0.8962	0.6801	0.5134
Echinops (FMNH 156189)	0.2023	0.8963	1.2650	0.5184	0.4479
Tenrec (FMNH 172708)	0.2930	0.8359	1.2375	0.6324	0.2625
Ptilocercus (FMNH 76855)	0.2523	0.7940	1.0547	0.5400	0.6848
Tupaia (FMNH 32994)	0.2480	0.8589	1.0197	0.6092	0.6846
Vulpes (FMNH 214921)	0.2848	0.8724	1.0468	0.6587	0.5275
Procyon (FMNH 221769)	0.2772	0.8965	0.9544	0.5812	0.6706

Mustela (FMNH 165361)	0.2599	0.8967	1.1233	0.5350	0.6900
Rhynchocyon (FMNH 151213)	0.1737	0.7854	0.9771	0.6583	0.5757
**Modern metatherians**	***0.2198***	***0.8249***	***0.9686***	***0.5628***	***0.5721***
Caenolestes (FMNH 124620)	0.2762	0.7965	0.8741	0.5066	0.6327
Lutreolina (FMNH 136826)	0.1927	0.8663	0.9435	0.5080	0.5665
Chironectes (FMNH 75093)	0.1891	0.8933	0.9841	0.5726	0.6169
Metachirus (FMNH 69323)	0.1968	0.8842	0.9577	0.6341	0.5410
Dasyurus (FMNH 127358)	0.2229	0.8124	0.9350	0.5629	0.6060
Didelphis (FMNH 152106)	0.2471	0.7648	1.0888	0.5923	0.4512
Monodelphis (FMNH 20251)	0.2136	0.7569	0.9969	0.5631	0.5905
**Overall mean**	***0.2400***	***0.8193***	***0.9624***	***0.5611***	***0.6043***
**Overall median**	***0.2446***	***0.8359***	***0.9577***	***0.5631***	***0.6191***
**Standard deviation**	***0.0646***	***0.0669***	***0.1333***	***0.0606***	***0.1181***

As with jaw dimensions, determining *x* and *z* coordinates for the SM and MP muscle origins on the skull could not be obtained by direct measurements of specimens, largely because 3D skull fossils of early mammals are extremely rare. Thus, the same specimens of modern and extinct mammals that were used for jaw dimension calculations were also used to determine approximate locations of the SM and MP origins (Supplementary Table S2). The SM origin was measured as the approximate location of the anterior zygoma, and the MP origin was measured as the center of the pterygoid process of the sphenoid bone. The distance between the anterior right zygoma and left zygoma was measured and divided by the width of the jaw. On average, the width between anterior zygomae is 96.24% of the jaw width (6 d.u.) (Supplementary Table S2), and therefore the width at the SM origins was set at 5.8 d.u., or 96.7% of the jaw width. Similarly, the distance from pterygoid process to pterygoid process is 24.0% of the width of the jaw, and therefore the width at the MP origins was set at 1.44 d.u., or 24.0% of the jaw width.

Because of the curvature of the TM around the braincase, the origin of the TM was not estimated from linear measurements of skulls. In the models, the TM force vector is directed posteriorly and slightly dorsally as it is in many extant mammals (Turnbull 1970). The TM is treated as a single muscle rather than being split into the anterior temporalis and posterior temporalis. However, merging the anterior and posterior regions of the temporalis as a single muscle vector is common in the literature (e.g. Turnbull 1970, Davis *et al.* 2010, Law *et al.* 2016). Further, the anterior and posterior portions of the TM contract concurrently in *Didelphis* (Crompton and Hylander 1986), and *Didelphis* is viewed as having the primitive condition of Triplet muscle groups that was likely present in the earliest therians (Weijs 1994, Crompton *et al.* 2011, Williams *et al.* 2011). Thus, separating the TM into different portions for the modeling analyses would be unlikely to have a significant effect on results.

Based on the various measurements and considerations discussed here, 3D coordinates were produced for muscle origins, muscle insertions, and jaw joints. Muscle insertion and jaw joint coordinates (*x, y, z*) are provided in Supplementary Figure S3, which includes the same jaw models as in Figure 6 except that individual models are shown for each mammal group. The coordinate system is based on that shown in Figure 2. The mandibular symphysis and muscle origin coordinates are kept constant in all models. The muscle origin coordinates used in the models are

not given in Supplementary Figure S3, but are provided here: SM (4.35, 1.1, 2.9), MP (1.75, 0.75, 0.72), and TM (-1.5, 2.9, -2.7). The working-side SM, working-side MP, and balancing-side TM are used in the model because these represent a Triplet muscle group that contracts synchronously during the power stroke of a chewing cycle (Weijs 1994). As previously discussed, the TM muscle vector is truncated due to the curvature of the braincase. Thus, the TM origin is not expected to reflect the true location of the muscle origin.

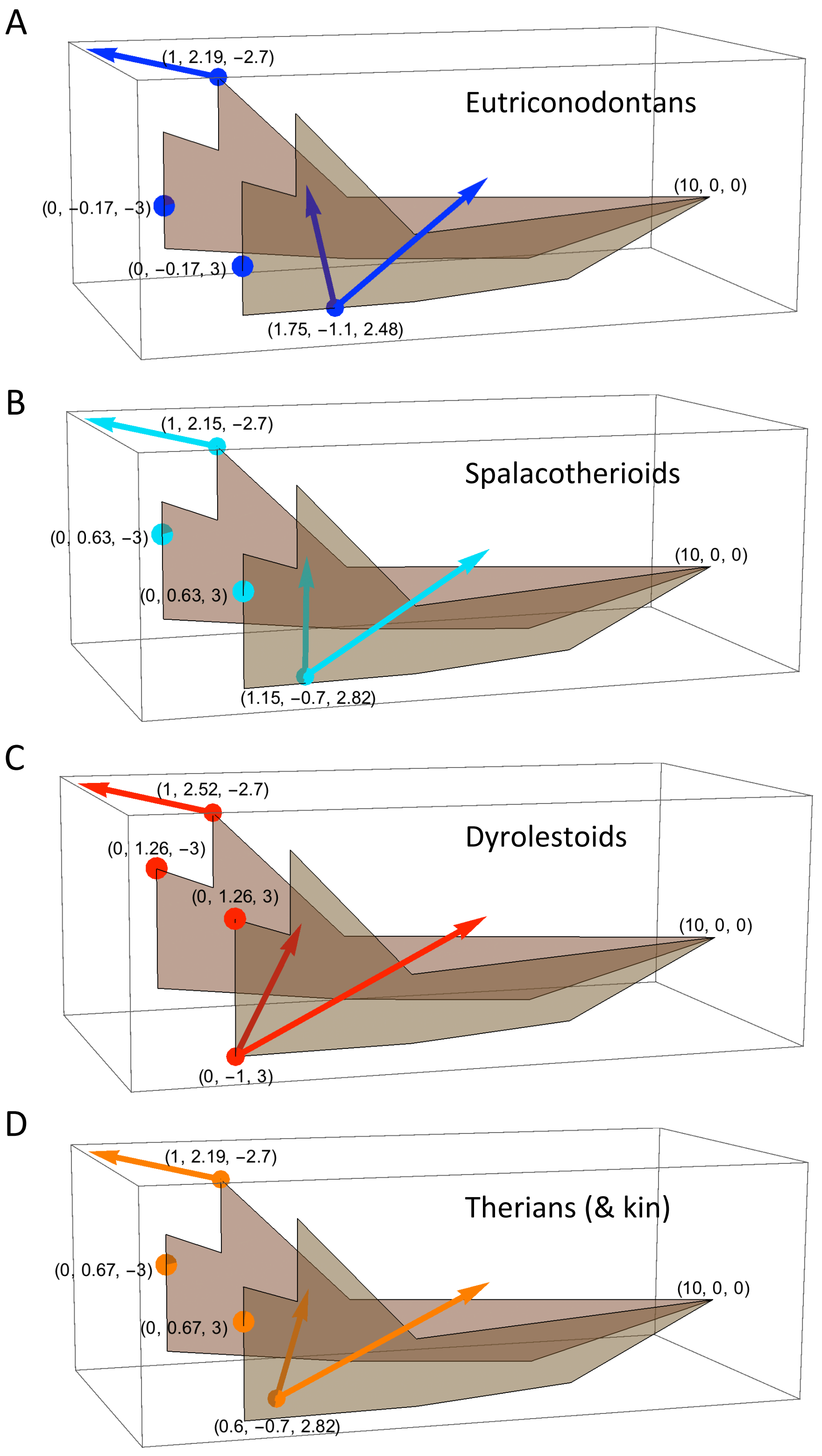

Supplementary Figure S3. Jaw models for early mammal groups with coordinates (*x, y, z*) for the jaw joints and muscle insertions. The SM and MP force vectors end at the muscle origins, the coordinates for which are provided in the text. Force vector lengths do not represent the relative strengths of the vectors. The *x, y,* and *z* coordinate system is based on that shown in Figure 2.

Muscle forces. Assigned muscle forces (*F* in Equation 1 of the main text) are based on relative muscle masses reported in Turnbull (1970), and these values are provided in the Methods and Figure 6 key. Turnbull expresses these as percentages of the overall mass of jaw musculature. Analyses are run with relative muscle masses for three mammalian species to help capture the range of relative muscle forces that might be found in early mammals. Thus, even if muscle masses are not perfect proxies for muscle forces, by including a variety of force values the analyses capture a range of potential results for the mammal groups. It is worth noting that results remain relatively similar when using the different force assignments (Fig. 6), suggesting that potential variation in muscle forces among mammal groups is unlikely to alter the broad trends seen in this study.

Rather than relative muscle masses, physiological cross-sectional area (PCSA) is often used in analyses of jaw mechanics to estimate muscle forces. PCSA is calculated using an equation that includes muscle mass, muscle density, fiber length, and fiber pennation angle (see Davis *et al.* 2010 and citations within). Unfortunately, this equation cannot be implemented in this study because information such as fiber lengths (for extinct taxa and most modern analogs) is unknown. However, a muscle density value near zero and a pennation angle of zero degrees are often used in the PCSA equation for all jaw muscles (e.g. Davis *et al.* 2010, Law *et al.* 2016). Thus, these factors should have little effect on calculations of PCSA, and muscle masses are expected to be an appropriate proxy for PCSA in this study. Alternatively, PCSA can be estimated based on the area of the infratemporal fossa. However, Davis *et al.* (2010) demonstrate that PCSAs based on infratemporal fossa area severely overestimate the contribution of the medial pterygoid and superficial masseter. Thus, estimates from muscle masses seem more appropriate.

Jaw models – moment calculations. The general method for calculating moment values for the musculoskeletal jaw configurations of early mammal groups is provided in the Methods section of the main text. This includes an explanation for why a single Triplet muscle group (as defined by Weijs 1994) is used for calculations (Figs. 2 and 6). However, some additional information is discussed here.

Moment calculations based on the jaw models of this study extend beyond conventional 2D jaw mechanics analyses by incorporating the third dimension (i.e., *z*-axis of Figure 2). Including the additional dimension is considerably beneficial by allowing for analyses of yaw rotation and roll rotation (Figs. 2 and 6). These analyses would not be possible if only 2D models based on lateral jaw images were used. However, it is worth noting that the *z*-axis values are estimated based on average measurements of many extant mammals and are kept constant among mammal groups. Thus, future studies should attempt to examine how variations in width of the jaw and muscle insertion/origin locations affect results (although see the Extended Results section for an analysis of variation in origin location), especially since these are likely to vary among early mammal groups.

Moment arm lengths are critical components of moment calculations. Moment arms are the perpendicular distances from individual force components to the axis of rotation. These are not visualized in the models of Figure 6 and Supplementary Figure S3. However, Figure 5 shows an example of moment arms (black lines, labeled *Lx* and *Ly*) in 2D for the eutriconodontan SM for pitch rotation around an axis through the jaw joints. The *x*- and *z*-axis components for the SM force

vector are also shown (gray arrows, labeled *Fx* and *Fy*), although they are scaled to the length of the muscle and not the size of the force vector. Moment arm lengths were calculated for all directional components of all muscle vectors. These are multiplied by the force components and then summed to calculate moment (i.e. torque) values (see Methods). See equations in the Methods section of the main text for further information on how these moment arm lengths were applied to calculate moment.

Mammals typically have unilateral mastication, meaning they have chewing cycles that include molars of a single hemimandible occluding with upper molars. Thus, only the working-side hemimandible needs to be considered when examining the power stroke of the chewing cycle in which molars are in occlusion. This is especially relevant for calculations of roll rotation, since only one hemimandible is used for this analysis (Fig. 6b).

Calculations of moment values can be simplified because not all force vector components contribute to rotation around axes. For instance, if the axis of rotation is mediolaterally oriented through both jaw joints (for pitch), the axis is parallel to the *z*-axis. In this case, the *x*- and *y*-axis force components contribute to the moment while the z-axis component does not. Hence, for calculations involving pitch (Fig. 6a), *z*-axis force components can be ignored. Similarly, *y*-axis force components do not contribute to yaw (Fig. 6b). Although the axis of rotation for roll is oblique for each group, the axis is rotated prior to moment calculations so that it falls along the x-axis (see below). Thus, the *x*-axis force component does not contribute to roll after rotation of the axis.

Moment calculations for roll rotation present a unique challenge in that the axes of rotation are oblique. The axis for roll is defined here as the line from the mandibular symphysis to the jaw joint, and this axis varies among mammal groups due to differences in jaw joint elevation (Fig. 6, Supplementary Table S3). For pitch and yaw, the axes of rotation are parallel to the *z*-axis and *y*-axis, respectively, and calculations can be simplified by only considering the two relevant force vector components, as described above. However, due to the oblique axis for roll, all three force components (i.e., *x*-, *y*-, and *z*-axis components) must be considered for roll analyses. To avoid the additional computation necessary for this analysis, I simply rotated the hemimandible model (and all associated points such as muscle origins) so that the axis of rotation was parallel to they *x*-axis. This included two rotations, which were performed using the RotationTransform function in Wolfram's Mathematica. First, the model was rotated horizontally so that all points on the jaw were in sagittal plane and had a z-axis value of zero. Second, the model was rotated vertically so that the axis of rotation was parallel to the *x*-axis. This second rotation was repeated individually for the models for each mammal group, since each group possessed a different axis of rotation that depends on the elevation of the jaw joint. After the rotations, roll rotation was calculated in the same manner as pitch and yaw.

Mechanical advantage. A commonly calculated performance metric for jaw mechanics is mechanical advantage. Mechanical advantage of a system is calculated as the in-lever (i.e., moment arm) length divided by the out-lever length. This can then be multiplied by the force vector components and summed for multiple muscles, as done for calculations of moment. For the jaw models in this study, the out-lever length would be the distance from the axis of rotation to the bite point. However, this measurement is disregarded here for several reasons: the molars are missing or

worn in many taxa; the elevation of the bite point could vary for molars that have wear facets of different elevations (e.g., tribosphenic molars have elevated trigonid cusps and a depressed talonid basin); and the relative length of the tooth row and position of the ultimate molar from the axes of rotation appear to be fairly consistent among mammal groups (see Extended Results, Supplementary Table S3), indicating that the out-lever distance would not change significantly among mammal groups. If the bite point is kept constant among mammal groups, the out-lever distance would remain the same for all calculations of yaw because the axes of rotation do not vary among groups (Fig. 6c). (Thus, 'moment' and 'mechanical advantage' are used interchangeably in the main text of this study.) However, the out-lever length will change in the calculations for pitch and roll since the axes of rotation are altered among mammal groups, although the effect of this change is expected to be minor. Therefore, future studies could explore the effects of this variable to a greater extent, especially for well-preserved taxa in which common bite points can be accurately identified. Results may also vary when mechanical advantage is calculated for other teeth besides the molars, such as the canines and incisors.

PART C. EXTENDED RESULTS AND DISCUSSION

Angular process (AP). Results of the AP geometric morphometrics (GM) analysis are shown in Figure 3. Results include three morphospace plots of the first two principal component analysis (PCA) axes, with PC1 along the horizontal axis and PC2 along the vertical axis. The three plots are identical but are replicated to highlight results for different mammal groups, which are designated by polygons. These plots are reproduced in Supplementary Figure S4 with labels for individual genera. As with Figure 3, the images are replicates of a single PCA plot for PC1 and PC2, but different mammal groups are highlighted by polygons in the replicates. These analyses were performed with semilandmark outlines of AP jaw regions, but see the Extended Methods and Supplementary Figures 1 and 2 for consideration of results when using sliding semilandmarks.

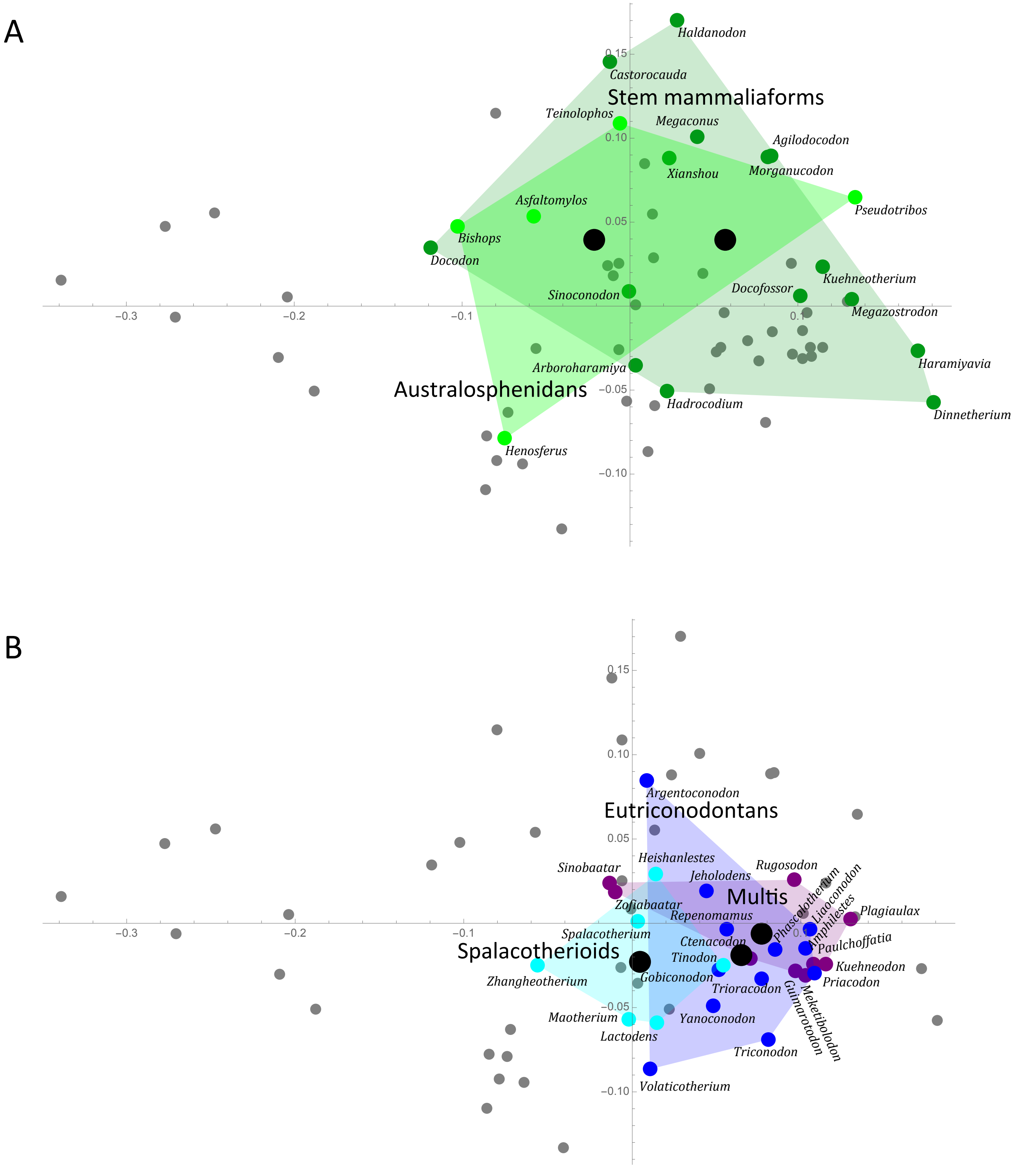
A
Haldanodon
Castorocauda
Stem mammaliaforms
Teinolophos
Megaconus
Agilodocodon
Xianshou
Morganucodon
Asfaltomylos
Pseudotribos
Bishops
Docodon
Kuehneotherium
Sinoconodon
Docofossor
Megazostrodon
Haramiyavia
Arboroharamiya
Australosphenidans
Hadrocodium
Dinnetherium
Henosferus
B
Argentoconodon
Eutriconodontans
Heishanlestes
Rugosodon
Sinobaatar
Jeholodens
Multis
Zofiabaatar
Repenomamus
Phascolotherium
Liaoconodon
Amphilestes
Plagiaulax
Spalacotherium
Spalacotherioids
Ctenacodon
Tinodon
Paulchoffatia
Kuehneodon
Zhangheotherium
Gobiconodon
Priacodon
Trioracodon
Guimarotodon
Meketibolodon
Maotherium
Yanoconodon
Lactodens
Triconodon
Volaticotherium

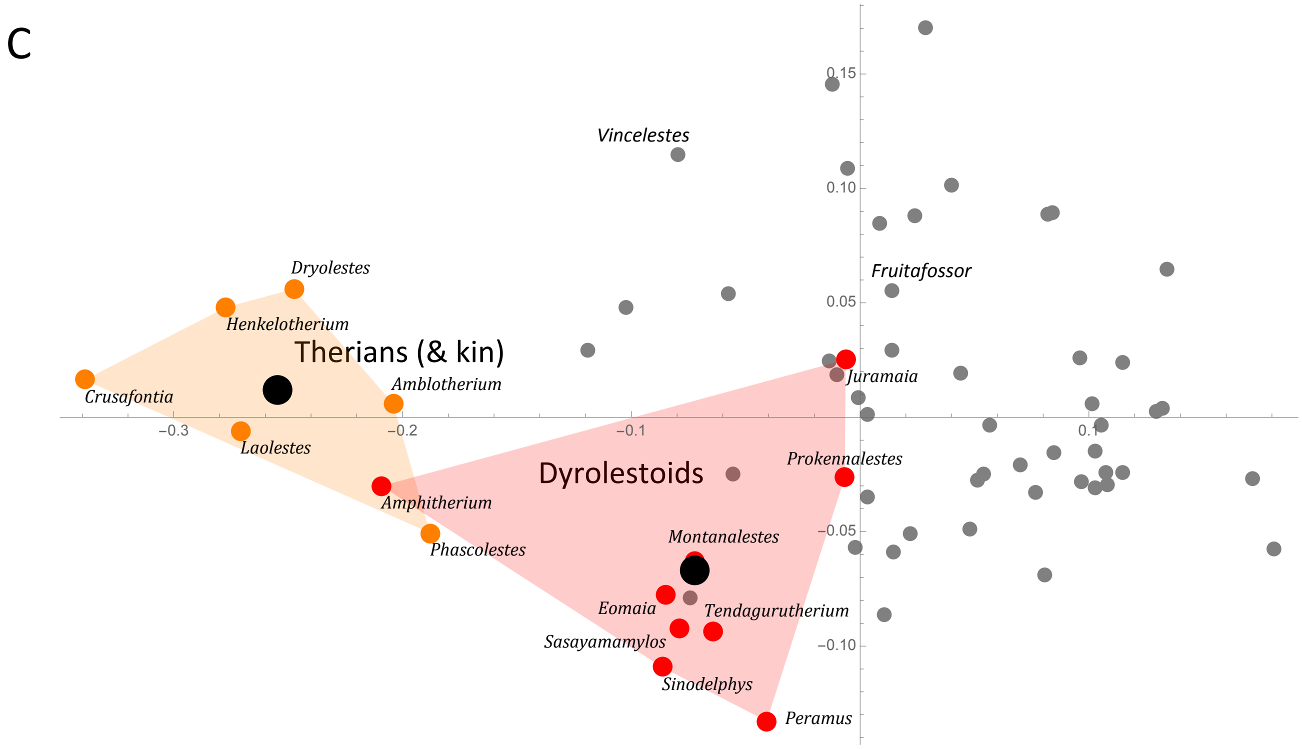

Supplementary Figure S4. The three PC1 and PC2 morphospace plots of AP shape that are shown in Figure 3, but with genera labeled. Large, black points represent the mean AP shape for each mammal group. The horizontal axis is PC1 (54.5% of variance), and the vertical axis is PC2 (17.4% of variance). *Vincelestes* and *Fruitafossor*, which are not included in a mammal group (see Mammal Groups section of the Extended Methods), are labeled in C. See the text and the Figure 3 caption for additional details. Abbreviation: multis, multituberculates.

A considerable shift in average jaw morphology occurs early in the crown mammalian tree. Compared to stem mammaliaforms (which have an anteriorly positioned AP), the most notable morphological change is the lack of an AP in three of the early crown mammal groups: multituberculates, eutriconodontans, and spalacotherioids (Fig. 3 and Supplementary Fig. S4). These are the ‘blue’ lineages in Figures 1, 3-6. However, it is worth noting that this lack of an AP does not necessarily indicate reduced medial pterygoid and superficial masseter muscles, since these taxa often possess deep fossae allowing for considerable muscle attachment.

At the cladotherian node, a second major shift in average jaw morphology occurs: cladotherians evolve a prominent, posterior AP. Cladotherians include dryolestoids and early therians, which are the ‘red’ lineages in Figures 1, 3-6. In dryolestoids (i.e., the earliest branching cladotherians), the distal end of the AP is located directly ventral to the jaw joint, whereas the APs in stem mammaliaforms are located in a position that is anterior to the jaw joint. Thus, dryolestoids do not overlap in PCA morphospace with earlier lineages that also possess an AP (Fig. 3 and Supplementary Fig. S4). Early therians also possess prominent APs, but they are not as posteriorly positioned as the APs of dryolestoids.

As noted in the main text, the results of the AP analysis support previous suggestions that the APs of non-mammalian cynodonts (which have been referred to as "pseudangular" processes) are not homologous to the APs of therians (e.g., Jenkins *et al.* 1983). This is in contrast to recent studies that have argued that the APs of non-mammalian cynodonts and mammals are homologous, based in part on the observation that the APs serve as insertion sites for the same jaw muscles in all groups (Abdala and Damiani 2004, Rougier *et al.* 2015). This debate over the homology of the AP seems to focus in part on whether the non-mammalian cynodont AP is homologous to the AP of the earliest crown mammals, australosphenidans. Here, the results support homology of the AP between these two groups, especially since they partially overlap in the GM morphospace (Fig. 3). This is in congruence with conclusions of Rougier *et al.* (2015). However, results of this study also conflict with conclusions of Rougier *et al.* (2015) by suggesting that the AP of australosphenidans and cladotherians are not homologous. Additional research and considerations may be needed to help resolve this issue.

Jaw joint elevation – implications. Eutriconodontans possess depressed jaw joints (Fig. 4, Supplementary Table S3). In addition, they lack an AP and have very little curvature of the posterior-ventral region of the jaw (Fig. 3). These traits are comparable to modern mammalian carnivores, which tend to possess a very reduced AP and a depressed condylar process. One eutriconodontan, *Repenomamus*, is a confirmed carnivore based on the presence of embryonic dinosaur bones found in the stomach region (Hu *et al.* 2005B). These results suggest that the jaw features of eutriconodontans evolved as adaptations for a carnivorous diet. Spalacotherioids tend to be smaller than eutriconodontans and therefore more likely to have been insectivores. Their jaw joints are less depressed than those of eutriconodontans and the posteroventral region of the lower jaw often forms a "bulge," although a true AP is not present (Figs. 3, 4). Thus, they appear to be morphological intermediates between eutriconodontans and cladotherians.

Supplementary Table S3. Jaw joint elevation, coronoid process elevation, and tooth row length of jaw specimens of this study (see Supplementary Table S1). The values are ratios, calculated by dividing the measurements by the length of the jaw. Median values for groups are in bold and italics. Jaw joint and coronoid process elevation results are shown in Figure 4. In the notes column, "molar landmark" refers to the single landmark between the penultimate and ultimate molars (Fig. 3), and "AP shape analysis" refers to the geometric morphometrics analysis of the angular process (AP) region of the jaw (Fig. 3).

Taxon	Jaw joint elevation	Coronoid process elevation	Tooth row length	Notes
**Stem Mammaliaforms**	***0.0493***	***0.2454***	***0.5808***	
Agilodocodon	0.1223	0.2458	0.6476	Part of the coronoid process is drawn with a dashed line in the source image and is likely to represent an estimate of is shape.
Arboroharamiya	0.0370	0.3997	0.5121	
Castorocauda	0.0242	0.1425	0.6642	Part of the coronoid process is drawn with a dashed line in the source image and is likely to represent an estimate of is shape.
Docodon	0.0265	0.2454	0.6702	The *Docodon victor* specimen from Simpson (1929) was used for all linear measurements but not for the AP shape analysis because the AP is likely incorrectly reconstructed (Rouger *et al.* 2015). The *Docodon apoxys* specimen described by Rougier *et al.* (2015) was used for the AP analysis, but it is missing a portion of the condylar process so it could not be used for linear measurements.
Docofossor	0.0749	0.3508	0.5855	
Dinnetherium	0.0279	0.1447	0.6020	
Hadrocodium	0.0617	0.1965	0.5023	
Haldanodon	0.1264	0.3068	0.5668	
Haramiyavia	0.0828	0.2538	0.5648	
Kuehneotherium	0.0983	0.1879	0.6475	
Megazostrodon	0.0493	0.1427	0.6191	Part of the coronoid process is drawn with a dashed line in the source image and is likely to represent an estimate of is shape.
Morganucodon	0.0834	0.2302	0.5808	
Sinoconodon	0.0283	0.2195	0.4843	A juvenile specimen (IVPP 4727) from Zhang *et al.* (1998) was used for jaw joint and tooth row measurements because more molars are present, but the adult specimen (IVPP 8688) appears to have similar dimensions.
Megaconus	0.0142	0.2519	0.5783	
Xianshou	0.0445	0.2543	0.4419	
**Australosphenidans**	***0.1179***	***0.2178***	***0.6462***	
Asfaltomylos	0.2118		0.5598	A portion of the AP is likely broken and in the incorrect position. I largely ignored the broken piece for the AP shape analysis. Jaw length is estimated because the anterior tip of the jaw is broken. The coronoid elevation is not measured because the coronoid process is not fully preserved or reconstructed.
Bishops	0.1989		0.6324	Jaw length is estimated because anterior tip of jaw is broken. The coronoid elevation is not measured because the coronoid process is not fully preserved or reconstructed.
Henosferus	0.0389	0.1988	0.6602	
Pseudotribos	0.0497	0.2369	0.6600	
Teinolophos	0.1179		0.7448	The coronoid elevation is not measured because the coronoid process is not fully preserved or reconstructed.
**Multituberculates**	***-0.0156***	***0.2558***	***0.4822***	
Ctenacodon	0.0856	0.2574	0.4822	
Guimarotodon	-0.0422	0.3054	0.5358	
Kuehneodon	-0.0031		0.4590	Molar landmark location is estimated due to lack of molars. The

				coronoid elevation is not measured because the coronoid process is not fully preserved or reconstructed.
Meketibolodon	0.0075	0.2283	0.4442	Part of the coronoid process is drawn with a dashed line in the source image and is likely to represent an estimate of is shape.
Paulchoffatia	-0.0159		0.4907	Molar landmark location is estimated due to lack of molars. The coronoid elevation is not measured because the coronoid process is not fully preserved or reconstructed.
Plagiaulax	0.0048	0.2692	0.4669	Molar landmark location is estimated due to lack of molars.
Rugosodon	-0.0860	0.1670	0.6464	
Sinobaatar	-0.0715	0.1720	0.6561	
Zofiabaatar	-0.0156	0.2558	0.4403	
**Eutriconodontans**	***-0.0174***	***0.2261***	***0.6340***	
Amphilestes	0.0263	0.2118	0.6798	
Argentoconodon	-0.0469	0.2449	0.7220	
Gobiconodon	-0.0462	0.2029	0.5308	
Jeholodens	0.0152	0.1230	0.7139	
Liaoconodon	0.0209	0.2891	0.6172	
Phascolotherium	0.0617	0.2426	0.5672	
Priacodon	-0.0087	0.2971	0.6586	
Repenomamus	-0.0379	0.2514	0.5350	
Triconodon	-0.0262	0.2335	0.5804	
Trioracodon	-0.0474	0.2186	0.6310	
Volaticotherium	-0.0586	0.1845	0.6370	
Yanoconodon	0.0150	0.2112	0.6448	
**Spalacotherioids (& *Tinodontids*)**	***0.0633***	***0.2215***	***0.6392***	
Heishanlestes	0.0633	0.2363	0.6392	Jaw length is estimated because anterior tip of jaw is broken.
Maotherium	0.0349	0.2015	0.5864	
Spalacotherium	0.1304	0.2401	0.6522	
Tinodon	0.0798	0.2215	0.6017	
Yermakia	0.0221	0.2370	0.6445	Not used in the AP shape analysis due to broken angular region. The jaw articulation point was estimated because the condylar process may be incomplete.
Zhangheotherium	-0.0042	0.1392	0.6123	
Lactodens	0.0952	0.1854	0.6858	
**Dryolestoids**	***0.1257***	***0.2515***	***0.6337***	
Amblotherium	0.0887	0.2196	0.6549	
Crusafontia	0.1056	0.2518	0.6709	
Dryolestes	0.1378	0.3167	0.5639	
Henkelotherium	0.1262	0.2512	0.6533	
Laolestes	0.1347	0.2469	0.6141	
Phascolestes	0.1252	0.2600	0.5868	
**Therians & close kin**	***0.0680***	***0.2191***	***0.6069***	
Eomaia	0.0480	0.2213	0.6675	
Juramaia	0.0693		0.6489	Some of the AP and condylar process shape was estimated by Luo *et al.* 2011. The coronoid elevation is not measured because the coronoid process is not fully preserved or reconstructed.
Montanalestes	0.0985		0.6069	Jaw length is estimated because anterior tip of jaw is broken. The coronoid elevation is not measured because the coronoid process is not fully preserved or reconstructed.
Peramus	0.0511	0.2191	0.5819	
Prokennalestes	0.0667	0.1341	0.5605	Jaw length is estimated because anterior tip of jaw appears broken. A small portion of the AP may also be broken.
Sasayamamylos	0.1133	0.3232	0.5876	
Sinodelphys	0.0636	0.1907	0.6781	Part of the coronoid process is drawn with a dashed line in the source image and is likely to represent an estimate of is shape.
Tendagurutherium				Only used in the AP analysis because the anterior portion of the jaw is missing (and therefore jaw length cannot be measured or

				estimated).
Amphitherium	0.1064	0.2150	0.5962	
**Other**				
Fruitafossor	0.1553	0.4217	0.5180	Not included with a mammal group because of its questionable phylogenetic affinity and derived features. See text.
Vincelestes	0.1423	0.1821	0.5055	Not included with a mammal group because of its questionable phylogenetic affinity and derived features. See text.

Jaws with joints at the same elevation as the molar row may be better adapted for slicing (analogous to scissors), and jaws with an elevated jaw joint may be better adapted for grinding by allowing multiple molars to occlude simultaneously (analogous to tongue-and-groove pliers) (Maynard Smith and Savage 1959, Greaves 1974). This concept may be relevant to early cladotherians due to the appearance of the talonid shelf of the molars. The very elevated jaw joints in dryolestoids (Fig. 4) may permit the paracones of multiple upper molars to occlude simultaneously with the talonid shelves of multiple lower molars as the jaw moves mediodorsally. However, if this was the sole reason for the elevated jaw joint then it could be expected that early therians, which possess grinding surfaces on talonid basins, would have jaw articulations with the same elevation, and this is not the case (Fig. 4). The jaw joint elevation in early therians is not as great as dryolestoids. Thus, additional considerations must account for the elevated jaw articulations of dryolestoids.

It is possible that the elevated jaw joint in dryolestoids is associated with the posterior position of the AP in this group (Fig. 3). Elevating the jaw joint provides space for the AP in the posteroventral region of the lower jaw, and it helps maintain the lengths of moment arms (i.e., in-levers) between the jaw joint (i.e., fulcrum) and force vectors of the SM and MP during pitch rotation.

Coronoid process. In this study, morphological changes to the coronoid process are emphasized to a lesser extent than changes to the AP. This is largely due to the observation that the major morphological change in early cladotherian jaws is the appearance of a prominent, posterior AP (Fig. 3). Hence, changes to the force vectors of MP and SM, which insert on the AP, are a major focus of this study. However, changes to the coronoid process (and insertion point for the TM) are considered by measuring the elevations of the coronoid process above the molar row (see Methods and Supplementary Extended Methods). The general conclusion is that the coronoid elevation does not vary considerably among groups, especially relative to changes in AP shape (Fig. 3) and jaw joint elevation (Fig. 4). Thus, the TM force vectors that are shown in Figure 6 (i.e., purple arrows) are kept constant for the four mammal groups in which models were produced. However, the slight differences in coronoid elevation among groups (Supplementary Table S3, Supplementary Fig. S3) are incorporated into the actual calculations of moment values. Average (median) coronoid elevation relative to jaw length for the early mammal groups: eutriconodontans, 22.6%; spalacotherioids, 21.5%; dryolestoids, 25.2%; and therians (and close kin), 21.9% (Supplementary Table S3). The most notable result is that dryolestoids have coronoid processes with greater elevations than those of additional mammal groups. This could be related to the elevated jaw joint

in this group, since a concurrent elevation of the coronoid process (and TM force vector) would help maintain the length of the moment arm for pitch rotation.

Further analyses of the coronoid process shape (such as a GM analysis that is similar to the one performed on AP shape) were not performed here for several reasons. First, the coronoid process in early mammal fossils is not as commonly preserved as the condylar or angular processes, meaning the sample is smaller for this jaw process (e.g., the australosphenidan group only includes coronoid elevation results for two genera). Second, because of the poor preservation of this process, the shape of the coronoid is often reconstructed with less confidence in publications (i.e., it is often drawn with a dashed line outline, indicating that the shape is uncertain). Third, there is considerable within-group variation in coronoid process shape. This makes it more difficult to discern broad evolutionary trends and blurs the differences between early mammal groups.

Tooth row length. In the jaw model analyses, the locations of the bite points are not incorporated. The importance of this variable becomes apparent when considering morphological and functional differences between pre-mammalian synapsids and mammaliaforms (Crompton and Hylander 1986). Pre-mammalian synapsids such as cynodonts tend to have shorter tooth rows (relative to total jaw length) than mammaliaforms. Thus, the typical bite points along the tooth row for cynodonts are expected to be more anteriorly located than the bite points of mammaliaforms. Variation in the bite point position is likely to alter the mechanical advantages and bite forces of different groups. For instance, calculation of mechanical advantage includes dividing the in-lever (i.e., moment arm) length by the out-lever length. The out-lever in the jaw models is the distance between the bite point and the axis of rotation, and therefore this value will vary among taxa with different bite point locations.

To help address whether bite point locations are expected to vary considerably among early mammal groups, I measured the relative tooth row lengths for the genera of this study (Supplementary Table S3). My assumption is that taxa with similar tooth row lengths will have similar bite point locations, on average, especially for the molars. Results are similar among the mammal groups for which I produced jaw models (Supplementary Table S3). Average (median) tooth row lengths relative to jaw length: eutriconodontans, 63.4%; spalacotherioids, 63.9%; dryolestoids, 63.4%; and therians (and close kin), 60.7% (Supplementary Table S3). Based on these results, the lengths between axes of rotation and the bite points (i.e., out-lever lengths) are not expected to vary considerably among mammal groups and are not examined in more detail.

Moment results. As the posterior processes of the lower jaw underwent evolutionary changes among early mammal groups, performance metrics (e.g., torque and mechanical advantage) for the musculoskeletal jaw configurations would have been altered for various movements. The 3D jaw models (Fig. 6, Supplementary Fig. S3) help assess differences in moment (i.e., torque or moment of force) for three types of jaw rotation (i.e., pitch, roll, and yaw). Results for these analyses are shown in Figure 6 and Supplementary Table S4. See the Results and Discussion section of the main text for discussion of the moment values for the mammal groups.

Supplementary Table S4. Moment values associated with the jaw model results in Figure 6. Results are given for the two yaw axes of rotation shown in Figure 6c. Results were calculated using multiple force assignments for the superficial masseter (SM), medial pterygoid (MP) and temporalis (TM) muscles (see text). These are based on the relative muscle weights reported by Turnbull (1970) for *Didelphis virginiana* (*D. v.*) (TM, 0.57; SM, 0.14; and MP, 0.07), *Echinosorex gymnurus* (*E. g.*) (TM, 0.61; SM, 0.11; and MP, 0.09), and *Canis familiaris* (*C. f.*) (TM, 0.67; SM, 0.10; and MP, 0.03).

	Pitch			Roll			Yaw (central)			Yaw (jaw joint)		
**Mammal Group**	***C. f.***	***D. v.***	***E. s.***	***C. f.***	***D. v.***	***E. s.***	***C. f.***	***D. v.***	***E. s.***	***C. f.***	***D. v.***	***E. s.***
Eutriconodontans	1.963	1.827	1.902	0.250	0.217	0.004	1.914	1.701	1.744	0.399	0.592	0.507
Spalacotherioids	1.425	1.368	1.403	0.239	0.199	-0.014	1.951	1.759	1.793	0.500	0.758	0.667
Dryolestoids	1.186	1.173	1.192	0.226	0.186	-0.017	2.040	1.875	1.900	0.600	0.924	0.830
Therians (& kin)	1.334	1.256	1.298	0.198	0.163	-0.014	1.989	1.818	1.842	0.576	0.883	0.789

Deep masseter. The deep masseter muscle is not included in functional analyses primarily because it is not considered a member of the Triplet muscle groups, as defined by Weijs (1994). Also, it does not insert on the jaw processes that are a focus of the morphometric analyses of this study, making it more difficult to examine evolutionary changes to the muscle vector. Further, estimation of deep masseter position in 3D is especially difficult without a distinct process for the muscle insertion (in contrast to the other muscles of this study) and preservation of the zygomatic arches for muscle origins.

Because the deep masseter is not one of the Triplet muscles, it is not expected to reach maximum contraction concurrently with the Triplet muscles. Instead of including the deep masseter with Triplet muscle groups, Weijs (1994) described the working-side deep masseter and balancing-side deep masseter as 'Vertical Closers.' However, Crompton *et al.* (2011) included the working-side deep masseter in their 'Group 1' muscles and balancing-side deep masseter in their 'Group 2' muscles for *Didelphis*, although they also note that the deep masseter is involved in the fast close and fast open stages of the chewing cycle. They depict Group 1 and Group 2 muscles contracting concurrently, similarly to the Triplet muscles of Weijs (1994). Thus, if the deep masseter was included in analyses, the balancing-side deep masseter would be included with the Triplet II muscles that are modeled in Figure 6.

Unlike the SM and MP, which have a large *x*-axis (horizontal) component (Figs. 5 and 6), the deep masseter is directed dorsolaterally (i.e., it has significant *z*- and *y*-axis components). The dorsally-directed component of the force vector is parallel to the axes of rotation during yaw, meaning that it does not affect yaw rotation. Similarly, the laterally-directed component is parallel to the axis of rotation during pitch, meaning that it does not affect pitch rotation. Thus, incorporation of the deep masseter into moment calculations for yaw and pitch may have a minimal effect on results for these types of rotation. Unlike yaw and pitch, the deep masseter may have a considerable effect on results for roll rotation (Fig. 6b). It connects the masseteric fossa of the jaw to the zygomatic arch, and the force vector likely has a relatively large moment arm for the axis of rotation for roll, especially if the zygomatic arch extends laterally from the skull. However, the deep masseter does not insert on the AP and is not expected to experience as great of change in vector as

that of the MP and SM among mammal groups. Therefore, including it in analyses is likely to alter results for all mammal groups in a similar manner, which is not expected to change the broad trends seen here.

As noted in the main text, some mammals such as carnivorans may produce transverse molar movements via mediolateral translation (along the *z*-axis of this study) rather than via yaw rotation. The deep masseter may be especially involved in this movement since it has a large lateral (i.e., *z*-axis) component. This is supported by results in Davis (2014), which show evidence of a late-contracting deep masseter causing significant mediolateral translation in the kinkajou.

Gape. The moment results from modeling analyses (Fig. 6) suggest that morphological jaw changes in early cladotherians (i.e., dryolestoids and early therians) may be adaptations associated with greater yaw rotation during mastication. However, it is possible that additional performance metrics played a role in the morphological changes in cladotherian jaws. For instance, musculoskeletal configurations of the jaw can have a considerable impact on gape, both in terms of the amount of gape allowed and the force produced by adductor muscles during varying degrees of gape. In general, the greater a muscle is put in tension (i.e., stretched), the greater the reduction in force output (Herring and Herring 1974). Therefore, muscles that stretch to a greater degree during jaw opening will produce less force during a wide gape. In the jaw models of this study (Fig. 6, Supplementary Fig. S3), the SM and MP are longer in dryolestoids and therians than in eutriconodontans and spalacotherioids due to the posteriorly extended AP. Thus, it is expected that the jaw muscles in early cladotherian would experience relatively less stretch of the muscle during jaw opening. This assumption suggests that increased gape and greater muscle forces during wide gape may be possible in early cladotherians, and that this may have been an additional factor in the evolution of an extended AP.

However, additional considerations indicate that gape is unlikely to have played a role in the evolution of an extended AP in early cladotherians. First, stem cladotherians were likely insectivores, based on their relatively small body sizes (especially compared to eutriconodontans), dentitions that are analogous to zalambdodont dentitions of modern "insectivorans" such as tenrecs, and jaw morphologies (Grossnickle and Polly 2013). Consuming insects is unlikely to require increased gape or strong forces during wide gape. Second, lengths and directions of muscle fibers affect force-tension curve of muscles and the amount of gape possible, and these variables are unknown for fossil taxa. Thus, additional analyses are necessary to further examine the effect of potential muscle fiber lengths and directions on gape in early mammals. Third, the AP of dryolestoids (and some insectivorans such as shrews) tends to be long and thin (Fig. 3), with the long axis of the AP pointed anteriorly in the direction of the inferred superficial masseter origin (near the anterior zygoma) during jaw closure (e.g., Turnbull 1970). Thus, the muscle force vectors are assumed to be largely parallel to the long axis of the AP (and mostly horizontal) when the teeth are in occlusion. However, during wide gape the posterior tip of the AP will rotate dorsally behind the jaw joint, bringing the muscle vectors of the SM and MP closer to the jaw joint and significantly reducing their moment arm lengths for pitch rotation (around an axis through the jaw joints). This suggests much weaker bite forces for pitch during wide gape. Also, as the AP rotates during wide gape its long axis is no longer pointed toward the muscle origins (instead it will point

ventromedially). If the SM and MP place a strong tensile force on this gracile AP while it is not aligned with (or parallel to) the direction of force, it is likely to put considerable strain on the AP.

Gape is more likely to have been a factor in the evolution of the eutriconodontan jaw morphology rather than the cladotherian jaw. For instance, modern taxa like carnivorans that require a wide gape for large food items tend to have a small AP that is positioned close to the jaw joint, and the jaw joint tends to be depressed (Herring and Herring 1974, Grossnickle and Polly 2013). Similarly, eutriconodontans have a depressed jaw joint, and the MP and SM insert close to the jaw joint. Eutriconodontans are some of the largest Mesozoic mammals and at least one genus, *Repenomamus*, has been shown to be carnivorous based on fossilized gut contents (Hu *et al.* 2005B). This convergent musculoskeletal configuration of carnivorans and eutriconodontans likely results in minimal muscle stretch during wide gape and/or maintains moment arm lengths for the muscles during pitch rotation (unlike taxa with a posteriorly extended AP), allowing a strong bite force to be maintained even during wide gape.

Jaws, middle ears, and macroevolution. The evolutionary transition that resulted in the origin of the definitive mammalian middle ear (DMME; i.e., a single dentary squamosal jaw joint and middle ear elements that are detached from the jaw) is somewhat paradoxical. That is, bones expected to receive considerable compression and tension at the jaw joint during mastication (i.e., the quadrate and articular bones) transitioned to delicate sound transmitting ossicles (i.e., the incus and malleus, respectively) in the middle ear. It has been suggested that in taxa with bones serving dual roles as jaw and ear components, musculoskeletal configurations must have minimized forces at the jaw joint while preserving strong bite forces (e.g., Crompton and Hylander 1986). Although a diversity of jaw morphologies satisfies these requirements (Reed *et al.* 2016), it is expected that some morphologies and functions would not be plausible. For example, an elevated jaw articulation relative to the molar row can result in greater reaction forces at the jaw joint (especially when muscle forces are anteriorly directed), meaning that a substantially elevated jaw joint is unlikely in taxa with dual functioning ear and jaw components (Reed *et al.* 2016). Thus, it has been hypothesized that constraints on musculoskeletal configurations of the jaw diminished when ear components were relieved of jaw joint functions (i.e., the dentary-squamosal joint became the sole jaw joint), resulting in a diversification of morphologies in early mammal groups (Crompton and Parker 1978). This hypothesis is supported by the observed morphological and taxonomic diversifications in Jurassic mammaliaforms (Fig. 1; Luo 2007, Grossnickle and Polly 2013, Close *et al.* 2015), in addition to the variety of jaw morphologies documented in this study (Figs. 3-4).

Although pre-mammaliaform cynodonts are not included in this study, it is worth considering their jaw and ear morphologies when examining macroevolutionary patterns of stem mammaliaforms and early crown mammals. The cynodont AP tends to be anteriorly positioned relative to that of crown mammals (e.g., Crompton and Hylander 1986). This is similar to stem mammaliaforms, which also have an anteriorly positioned AP. However, unlike stem mammaliaforms, the AP is ventrally deeper (due to a ventrally extended AP and/or a deeper mandibular body) on average. An issue with an anteriorly positioned AP is that the attached adductor muscles may impede gape, since muscle stretch will be greater (if all other variables are kept constant) when the muscles are further from the axis of rotation. By extending the angular

process ventrally, cynodonts lengthen the jaw adductor muscles and likely lessen the forces lost to stretched muscles during gape. In addition, cynodonts appear to have a shortened tooth row relative to the length of the jaw (e.g., Crompton and Hylander 1986), which means the typical bite point (of molars) is expected to be more anteriorly positioned relative to that of mammaliaforms. The combination of the anterior AP and anterior bite point may have helped lessen the reactionary forces at the jaw joint during pitch rotation (Crompton and Hylander 1986, Reed *et al.* 2016).

In stem mammaliaforms in which the ear elements no longer perform a load-bearing function at the jaw joint, evolutionary constraints on the musculoskeletal configurations of the jaw may have been reduced. As noted above, this may have allowed for jaw joints that were significantly elevated. Further, it may have permitted jaw configurations with greater reactionary forces on the jaw joint because the delicate ear elements were no longer attached. Thus, the bite point and AP could move posteriorly, likely raising reactionary forces but benefitting the taxa in additional ways (e.g., allowing for a longer tooth row). See Crompton and Hylander (1986) and Reed *et al.* (2016) for additional considerations.

Early crown mammals such as eutriconodontans and spalacotherioids possess a bony connection between the middle ear and jaw via an ossified Meckel's cartilage (Fig. 1; Wang *et al.* 2001, Meng *et al.* 2003, Luo *et al.* 2007A, Ji *et al.* 2009, Luo 2011), but this connection does not appear to be present in early cladotherians. It is worth noting that mandibles of early cladotherians often possess a Meckel's groove (e.g., Davis 2012, Close *et al.* 2016), indicating that a cartilaginous ear-jaw connection may be maintained in adults. However, the lack of fossil evidence for a strong ear-jaw attachment suggests that any connection that remained was not rigid. Thus, the possible loss of a strong attachment between the jaw and middle ear in cladotherians may have permitted further diversification of jaw morphologies and functions, although this is speculative due to the limited fossil evidence.

Chewing cycles of stem mammaliaforms and early mammals undoubtedly involved significant pitch rotation to produce orthal occlusion. Since middle ears remain attached to the jaws of many of these groups (including spalacotherioids and eutriconodontans), it suggests that the presence of attached middle ears did not hinder pitch rotation. However, this may not be the case for yaw rotation. During yaw rotation, the condylar processes must protract and retract relative to the glenoid fossa (Fig. 2). Rather than simply rotating the middle ear elements, as occurs during pitch, protraction during yaw might create tension in the ear elements and the retraction during yaw might put stress on the ear elements. Thus, I posit that significant yaw rotation is likely in jaws that maintain a solid attachment to the middle ear. However, additional studies and fossil discoveries are necessary to further examine this hypothesis.

Cladotherian evolution. Mammaliaforms experienced an evolutionary radiation in the Jurassic that was marked by the appearance and diversification of numerous clades (Fig. 1; Luo 2007, Grossnickle and Polly 2013, Close *et al.* 2015). Most of these groups persisted for tens of millions of years and achieved considerable ecomorphological diversity (e.g., Luo 2007, Grossnickle and Polly 2013, Meng *et al.* 2015, Chen and Wilson 2015). However, many mammaliaform and early crown mammalian clades went extinct or were greatly diminished during the Cretaceous Terrestrial Revolution (KTR) at ~125-80 million years ago (Ma) and K-Pg mass

extinction event at 66 Ma, periods of considerable environmental perturbation and ecological change (e.g., Alvarez *et al.* 1980, Labandeira et al. 2002, Lloyd *et al.* 2008, Bond and Scott 2010, Tobin *et al.* 2012, Grossnickle and Polly 2013). Cladotherians survived the KTR and K-Pg extinction event and, led by therians, diversified after both events (Alroy 1996, Benson *et al.* 2013, Grossnickle and Polly 2013, Wilson 2014, Grossnickle and Newham 2016). Cladotheria includes "eupantotherians" (comprised primarily of Dryolestida, Amphitheriida, and "peramurans"), which were abundant in the Late Jurassic and regionally diverse in South America in the Cretaceous, and therians that began a diversification event in the Late Cretaceous (Grossnickle and Newham 2016) and now comprise all but five species of living mammals. Thus, cladotherians have been globally diverse for over 150 million years, and examining their early history is critical to understanding the origins of modern mammalian diversity.

The masticatory changes in early cladotherians that are described in this study may have resulted in greater occlusal precision, more efficient food processing, and greater dietary diversity. For instance, the appearance of a crushing function in the tribosphenic molars of therians may have allowed for improved mastication of plant matter. Thus, this consideration offers a possible explanation for the differential survival of cladotherians during periods of ecological perturbations and elevated extinction rates, such as the KTR and K-Pg extinction event. This conclusion is supported by evidence suggesting that early mammals with generalist diets were less prone to extinction than dietary specialists (Simpson 1944, Smits 2015, Grossnickle and Newham 2016). However, since diet is not directly tested in this study, further examination is needed to test this hypothesis.

Australosphenidans. Australosphenidans are not the focus of this study, due in part to a scarcity of fossils, questions concerning their phylogenetic affinities (see the Mammal Groups section of the Extended Methods), and the dominance of modern mammalian faunas by therians. However, morphological results for this group do lend some support to the hypothesized link between molar morphology, jaw morphology, and jaw yaw. Some australosphenidans possess jaw morphologies that are similar to those of therians (Figs. 3 and 4, Supplementary Table S3). Further, australosphenidans are believed to have convergently evolved tribosphenic molar morphologies and detachment of the middle ear from the jaw (Luo *et al.* 2002, Ramírez-Chaves al 2016). This suggests convergent adaptations in these clades, and it supports the predicted functional link between jaw, molar, and ear morphologies. It is worth noting that the only additional extant mammalian group to survive the KTR and K-Pg extinction events is Australosphenida (including monotremes), providing support to the hypothesis that the evolutionary changes to the molars, jaws, ears, and chewing cycles may have assisted in mammalian survival during these events.

Docodonts. Docodonts are a diverse group of mammaliaforms that are included within the 'stem mammaliaforms' group of this study. They are an additional group (besides therians and australosphenidans) with tribosphenic-like molars that suggest mediolateral movement during occlusion (e.g., Pfretzschner et al. 2005). They often have a distinct AP (e.g., Rougier *et al.* 2015), although it is not as posteriorly positioned as that of early therians.

In some respects, the results for docodonts conflict with the conclusions of this study. For instance, the molar morphologies suggest mediolateral movement during occlusion (Pfretzschner et

al. 2005) like that seen in early cladotherians, but the jaw joints are not as elevated and the AP is not as posteriorly positioned as those of early cladotherians. Thus, the musculoskeletal configurations in docodonts are not expected to be as ideal for yaw, at least in comparison to early cladotherians. However, there are a couple considerations that may help explain these conflicting results. First, docodont jaws maintain attached middle ear elements, which may inhibit the evolution of potential jaw morphologies. For instance, the middle ear elements may prohibit posterior migration of the angular process, and if taxa need to maintain small resultant forces at the jaw joint region because of attached ear elements then a depressed jaw joint may be necessary (Crompton and Hylander 1986). Thus, if docodonts are experiencing yaw rotation during occlusion, ideal musculoskeletal jaw configurations (like those in early cladotherians) may simply not be possible due to additional factors. Second, there is a possibility that the transverse molar movements in docodonts are produced by mediolateral translation (along the *z*-axis) rather than by yaw. For instance, the molar morphologies of docodonts are complex in shape and may direct molar movement during occlusion, meaning that jaw muscle control of yaw may not be necessary. Prominent X and Y cusps (of the medial portion of the upper molars) appear to truncate medial movement of lower molars during occlusion (Pfretzschner et al. 2005). Also, the medial region of the upper molars (analogous to the trigon of tribosphenic molars) is often directed posteromedially (e.g. Luo and Martin 2007). This is in contrast to early cladotherians and tribosphenic taxa in which the trigon is often directed medially or anteromedially. This suggests that a relatively unique type of occlusal movement may be occurring in docodonts, such as posteromedial movement of lower molars (Gingerich 1973). For instance, yaw rotation may be occurring around a vertical axis of rotation that is at or near the working side jaw joint (rather than the balancing side jaw joint as modeled in Figure 6), and yaw around an axis in this position was not tested in this study. Thus, the docodont musculoskeletal jaw configuration may be more ideal for yaw around an axis in this position, although this possibility will need to be explored in future studies.

Muscle origins – sensitivity test. The focus of the jaw model analyses is on evolutionary changes to muscle insertion locations and jaw joint elevations (Figs. 2-6). However, an additional variable that is expected have a considerable effect on muscle vectors and moments is the locations of muscle origins. Muscle origin locations are based primarily on measurements of skulls of modern analogs and well-preserved mammaliaform skulls (see Extended Methods and Supplementary Table S2), and these locations are kept constant in the jaw models of Figure 6 and Supplementary Figure S3.

It is possible that concurrent evolutionary changes to muscle insertion and muscle origin locations would negate the changes in moment values that are reported here. For instance, the SM muscle vector angles in Figure 5 are shown as being closer to horizontal in dryolestoids (in comparison to eutriconodontans and spalacotherioids) due to the posterior extension of the AP. However, if the SM origin evolved posteriorly with the concurrent evolution of a more posterior muscle insertion, this would negate the inferred change in muscle vector angle (Figs. 5 and 6). This could alter the moment value trends reported in Figure 6.

To examine the effect of potential variation in muscle origin locations on moment calculations, a sensitivity test was performed. This involved altering muscle origin locations for the

three muscles of this study (i.e., the working-side SM, working-side MP, and balancing-side TM) and repeating moment value calculations. The SM and MP muscle origins were altered along the *x*-axis and *y*-axis, and the TM was altered along the *y*-axis and *z*-axis (Supplementary Fig. S5). For the SM and MP, muscle origins were shifted two standard deviations in both directions away from the original origin location (Fig. 6, Supplementary Fig. S3). The standard deviations are those reported in Supplementary Table S2 and are therefore based on variation seen in modern analogs and fossil skulls. Since the locations were moved two standard deviations, this means that approximately 95% of the variation seen in these taxa is captured in the analyses. For the TM, muscle origin location was not based on measurements of skulls (see Extended Methods), so the muscle vector was moved approximately the same amount in each direction as was done for the SM (Supplementary Fig. S5). Muscle force assignments in all calculations are based on *Didelphis* muscle masses (see Methods and Extended Methods). Moment calculations were repeatedly calculated using the same methodology as described previously (see the Methods and Extended Methods). However, roll rotation was excluded because results for roll remained low in all previous calculations (Fig. 6b) and is not expected to change significantly with different muscle origin locations, especially since the TM is not included in roll calculations (see main text). Results for the muscle origin sensitivity test are provided in Supplementary Figure S5, with model images for spalacotherioids shown with muscle vectors based on the varying muscle origins (i.e., multiple blue arrows from the same muscle insertion location).

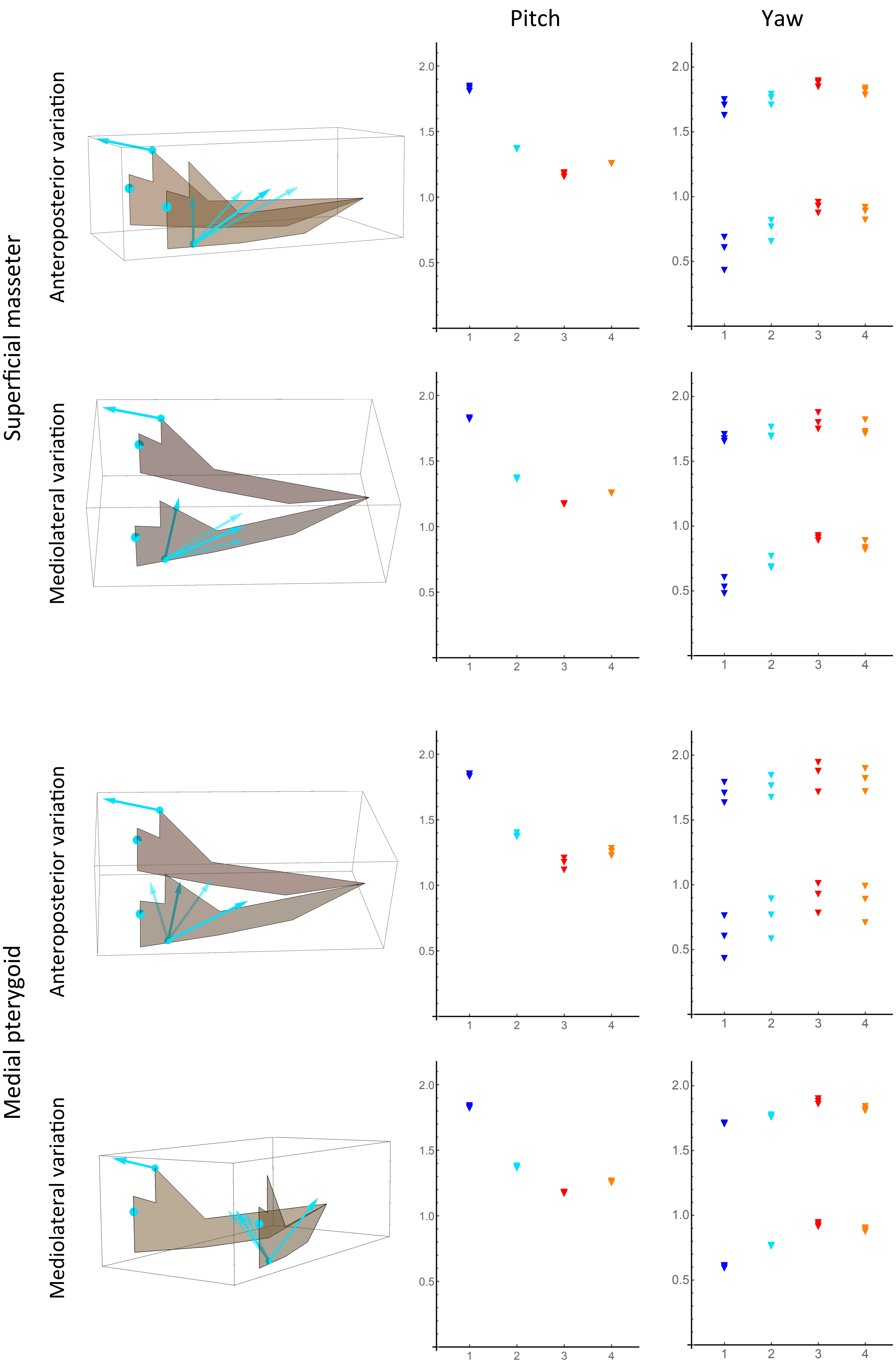
Pitch
Yaw
Superficial masseter
Anteroposterior variation
Mediolateral variation
Medial pterygoid
Anteroposterior variation
Mediolateral variation
2.0
1.5
1.0
0.5
1
2
3
4

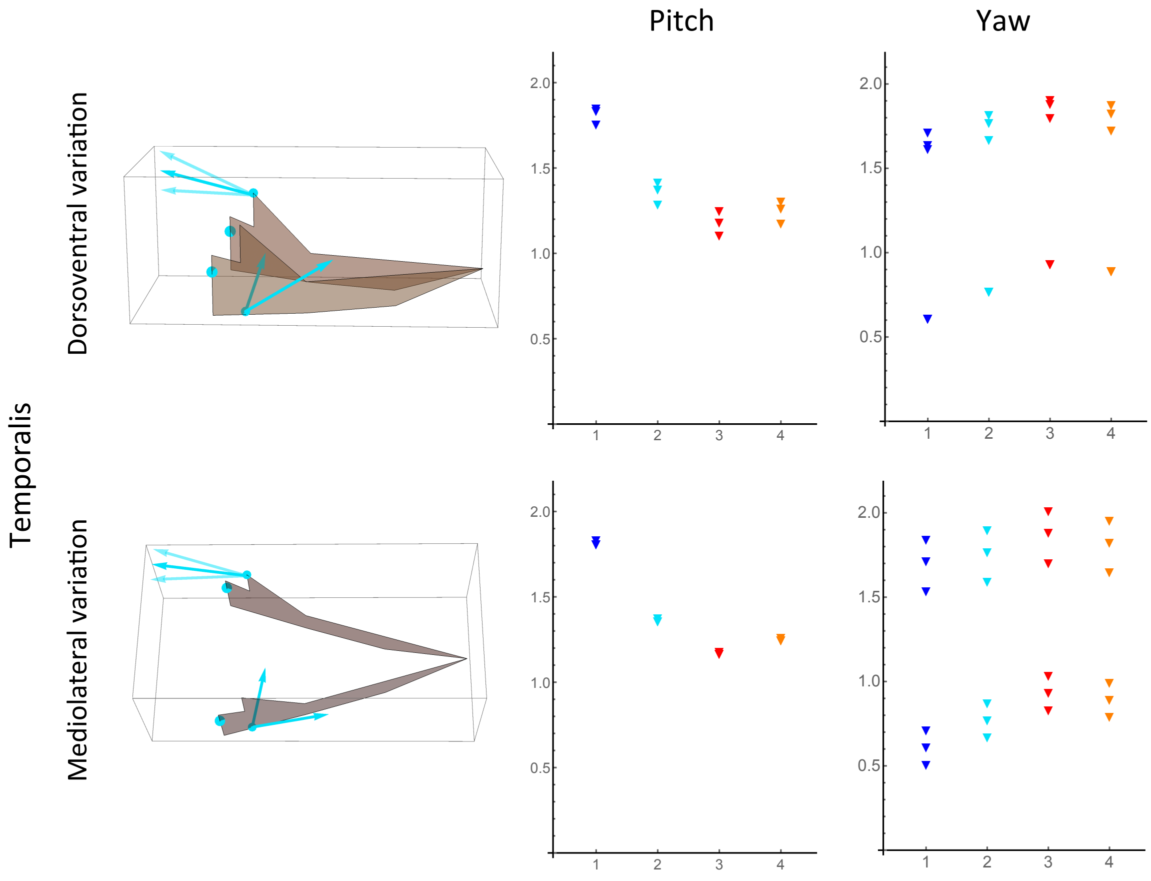

Supplementary Figure S5. Sensitivity test in which muscle origin locations have been altered to examine variation associated with potential evolutionary changes to these locations. The model images (left) are spalacotherioids, although moment analyses (right) were performed for the four mammal groups: eutriconodontans (blue, 1), spalacotherioids (cyan, 2), dryolestoids (red, 3); and therians and close kin (orange, 4). For the superficial masseter and medial pterygoid, origin locations were moved two standard deviations in both directions from the original muscle origin, and these new vectors are denoted with additional arrows in the model images. (The standard deviations are based on results of measurements in Supplementary Table S2). Only pitch and yaw were examined (see text), and the muscle force assignments in all analyses are based on muscle masses of *Didelphis*. As in analyses shown in Figure 6, there are two sets of results for yaw that are based on two potential locations for the axis of rotation. The greater values always correspond to the vertical axis that is just medial to the balancing side jaw joint. See Figure 6 for locations of these axes and additional information.

In the original moment calculations, early cladotherians (i.e., dryolestoids and therians) show relatively small moments for pitch and relatively large moments for yaw (Fig. 6). This pattern remains in all analyses of the sensitivity test in which muscle origin locations are altered (Fig. S5). In addition, for some calculations (e.g., mediolateral variation of the MP) the results for pitch and yaw remain nearly unchanged when the origin location is altered.

Results for pitch rotation are especially consistent for all calculations of the sensitivity test (Supplementary Fig. S5). This suggests that the pattern shown for pitch in the original analysis (Fig. 6) is unlikely to have been altered by evolutionary changes to the muscle origin locations among mammal groups. This is likely due to pitch results being based largely on jaw joint elevation and coronoid process elevation, since the distance between these points roughly represents the moment arm length for the large TM during pitch. Thus, changes to the angle of the TM force vector are unlikely to alter pitch results significantly unless the TM vector changes considerably more than the amount that is tested here. This provides additional evidence for the conclusion that musculoskeletal jaw configurations of early cladotherians were less ideal for pitch rotation than those of eutriconodontans and spalacotherioids.

Compared to pitch, results for yaw show greater variation when muscle origin locations are altered (Supplementary Fig. S5). However, this variation is still minimal for many of the calculations. The greatest variation is for anteroposterior changes to the MP origin and mediolateral changes to the TM origin. Thus, moment results for yaw in Figure 6 should be considered with some caution, although it is worth reiterating that the general trends for the four mammal groups are not altered when the muscle origins are varied. Considerable evolutionary change in muscle origin would have had to occur among early mammal groups to disrupt the original results pattern from Figure 6.

The sensitivity analysis tests the variation in muscle origin locations of modern taxa, but it cannot be ruled out that early mammal groups had muscle origin locations that are outside of the range of variation seen in modern analogs (and fossil mammals) that were measured for this study (Supplementary Table S2). However, there is little or no evidence to suggest that this is the case for these taxa, especially since the skull material of early mammals in this study (e.g., *Yanoconodon, Maotherium, Juramaia*, *Vincelestes*, *Eomaia*, *Sinodelphys*) do not indicate significant morphological divergence beyond the variation seen in modern analogs.

PART D. SUPPLEMENTARY INFORMATION REFERENCES

Abdala, F., & Damiani, R. (2004). Early development of the mammalian superficial masseter muscle in cynodonts. *Palaeontologia Africana*, *40*, 23-29.

Adams, D. C., & Otárola-Castillo, E. (2013). geomorph: an R package for the collection and analysis of geometric morphometric shape data. *Methods in Ecology and Evolution*, *4*(4), 393-399.

Alroy, J. (1996). Constant extinction, constrained diversification, and uncoordinated stasis in North American mammals. *Palaeogeography, Palaeoclimatology, Palaeoecology*, *127*(1), 285-311.

Alvarez, L. W., Alvarez, W., Asaro, F., & Michel, H. V. (1980). Extraterrestrial cause for the Cretaceous-Tertiary extinction. *Science*, *208*(4448), 1095-1108.

Bakker, R. T., & Carpenter, K. (1990). A new latest Jurassic vertebrate fauna, from the highest levels of the Morrison Formation at Como Bluff, Wyoming. Part III. The mammals: a new multituberculate and a new paurodont. *Hunteria*, *2*(6), 4-8.

Benson, R. B., Mannion, P. D., Butler, R. J., Upchurch, P., Goswami, A., & Evans, S. E. (2013). Cretaceous tetrapod fossil record sampling and faunal turnover: implications for biogeography and the rise of modern clades.*Palaeogeography, Palaeoclimatology, Palaeoecology*, *372*, 88-107.

Bi, S., Wang, Y., Guan, J., Sheng, X., & Meng, J. (2014). Three new Jurassic euharamiyidan species reinforce early divergence of mammals. *Nature*, *514*(7524), 579-584.

Bonaparte, J. F., & Rougier, G. W. (1987). Mamiferos del Cretacico inferior de Patagonia. *IV Congreso Latinamericano de Paleontología*, *1*, 343-359.

Bond, W. J., & Scott, A. C. (2010). Fire and the spread of flowering plants in the Cretaceous. *New Phytologist*, *188*(4), 1137-1150.

Bookstein, F. L. (1997). *Morphometric tools for landmark data: geometry and biology*. Cambridge University Press.

Chen, M., & Wilson, G. P. (2015). A multivariate approach to infer locomotor modes in Mesozoic mammals. *Paleobiology*, *41*(02), 280-312.

Cifelli, R. L. (1999). Tribosphenic mammal from the North American early Cretaceous. *Nature*, *401*(6751), 363-366.

Close, R. A., Friedman, M., Lloyd, G. T., & Benson, R. B. (2015). Evidence for a mid-Jurassic adaptive radiation in mammals. *Current Biology*, *25*(16), 2137-2142.

Crompton, A. W., & Jenkins, F. A. (1968). Molar occlusion in Late Triassic mammals. *Biological Reviews*, *43*(4), 427-458.

Crompton, A. W., & Parker, P. (1978). Evolution of the Mammalian Masticatory Apparatus: The fossil record shows how mammals evolved both complex chewing mechanisms and an effective middle ear, two structures that distinguish them from reptiles. *American Scientist*, *66*(2), 192-201.

Crompton, A. W., & Hylander, W. L. (1986). Changes in mandibular function following the acquisition of a dentary-squamosal jaw articulation. In Hotton, N., III, MacLean, P. D., Roth, J. J., & Roth, E. C. (eds.), *The ecology and biology of mammal-like reptiles*, 263-282. Smithsonian Institution Press, Washington, D. C.

Crompton, A. W. (2011). Masticatory motor programs in Australian herbivorous mammals: Diprotodontia. *Integrative and comparative biology*, *51*(2), 271-281.

Davis, J. S. (2014). *Functional Morphology of Mastication in Musteloid Carnivorans* (Doctoral dissertation, Ohio University).

Davis, J. L., Santana, S. E., Dumont, E. R., & Grosse, I. R. (2010). Predicting bite force in mammals: two-dimensional versus three-dimensional lever models. *Journal of Experimental Biology*, *213*(11), 1844-1851.

Davis, B. M. (2011). Evolution of the tribosphenic molar pattern in early mammals, with comments on the "dual-origin" hypothesis. *Journal of Mammalian Evolution*, *18*(4), 227-244.

Davis, B. M. (2012). Micro-computed tomography reveals a diversity of Peramuran mammals from the Purbeck Group (Berriasian) of England.*Palaeontology*, *55*(4), 789-817.

De Muizon, C. (1998). Mayulestes ferox, a borhyaenoid (Metatheria, Mammalia) from the early Palaeocene of Bolivia. Phylogenetic and palaeobiologic implications. *Geodiversitas*, *20*(1), 19-142.

Gaetano, L. C., & Rougier, G. W. (2011). New materials of Argentoconodon fariasorum (Mammaliaformes, Triconodontidae) from the Jurassic of Argentina and its bearing on triconodont phylogeny. *Journal of Vertebrate Paleontology*, *31*(4), 829-843.

Gambaryan, P. P., & Kielan-Jaworowska, Z. (1995). Masticatory musculature of Asian taeniolabidoid multituberculate mammals. *Acta Palaeontologica Polonica*, *40*(1), 45-108.

Gill, P. G., Purnell, M. A., Crumpton, N., Brown, K. R., Gostling, N. J., Stampanoni, M., & Rayfield, E. J. (2014). Dietary specializations and diversity in feeding ecology of the earliest stem mammals. *Nature*, *512*(7514), 303-305.

Gingerich, P. D. (1973). Molar occlusion and function in the Jurassic mammal Docodon. *Journal of Mammalogy*, *54*(4), 1008-1013.

Gunz, P., Mitteroecker, P., & Bookstein, F. L. (2005). Semilandmarks in three dimensions. In *Modern morphometrics in physical anthropology*, 73-98. Springer US.

Greaves, W. S. (1974). Functional implications of mammalian jaw joint position. *Forma et functio*, *7*, 363-376.

Grossnickle, D. M., & Newham, E. (2016). Therian mammals experience an ecomorphological radiation during the Late Cretaceous and selective extinction at the K–Pg boundary. *Proceedings of the Royal Society of London B: Biological Sciences, 283* (1832), 20160256.

Grossnickle, D. M., & Polly, P. D. (2013). Mammal disparity decreases during the Cretaceous angiosperm radiation. *Proceedings of the Royal Society of London B: Biological Sciences*, *280*(1771), 20132110.

Hahn, G. (1969). Beiträge zur Fauna der Grube Guimarota Nr. 3. Die Multituberculata. *Palaeontographica Abteilung A*, *133*, 1-100.

Hahn, G., & Hahn, R. (1998). Neue Beobachtungen an Plagiaulacoidea (Multituberculata) des Ober-Juras 2. Zum Bau des Unterkiefers und des Gebisses bei Meketibolodon und bei Guimarotodon. *Berl Geowiss Abh E*, *28*, 9-37.

Han, G., & Meng, J. (2016). A new spalacolestine mammal from the Early Cretaceous Jehol Biota and implications for the morphology, phylogeny, and palaeobiology of Laurasian 'symmetrodontans'. *Zoological Journal of the Linnean Society*, *178*(2), 343-380.

Heinrich, W. D. (1998). Late Jurassic mammals from Tendaguru, Tanzania, East Africa. *Journal of Mammalian Evolution*, *5*(4), 269-290.

Henkel, S., & Krebs, B. (1977). Der erste Fund eines Säugetier-Skelettes aus der Jura-zeit. *Umschau in Wissenschaft und Technik*, *77*(7), 217-218.

Herring, S. W., & Herring, S. E. (1974). The superficial masseter and gape in mammals. *American Naturalist*, 561-576.

Hu, Y., Fox, R. C., Wang, Y., & Li, C. (2005A). A new spalacotheriid symmetrodont from the Early Cretaceous of northeastern China. *American Museum Novitates, 3475,* 1-20.

Hu, Y., Meng, J., Wang, Y., & Li, C. (2005B). Large Mesozoic mammals fed on young dinosaurs. *Nature*, *433*(7022), 149-152.

Hu, Y., & Wang, Y. (2002). Sinobaatar gen. nov.: first multituberculate from the Jehol Biota of Liaoning, northeast China. *Chinese Science Bulletin*, *47*(11), 933-938.

Hu, Y., Wang, Y., Luo, Z.-X., & Li, C. (1997). A new symmetrodont mammal from China and its implications for mammalian evolution. *Nature*, *390*(6656), 137-142.

Jenkins, F. A., Crompton, A. W., & Downs, W. R. (1983). Mesozoic mammals from Arizona: new evidence on mammalian evolution. *Science*, *222*(4629), 1233-1235.

Jenkins Jr, F. A., & Schaff, C. R. (1988). The Early Cretaceous mammal Gobiconodon (Mammalia, Triconodonta) from the Cloverly Formation in Montana. *Journal of Vertebrate Paleontology*, *8*(1), 1-24.

Ji, Q., Luo, Z. X., Ji, S. A. (1999). A Chinese triconodont mammal and mosaic evolution of the mammalian skeleton. *Nature*, *398*(6725), 326-330.

Ji, Q., Luo, Z. X., Yuan, C. X., & Tabrum, A. R. (2006). A swimming mammaliaform from the Middle Jurassic and ecomorphological diversification of early mammals. *Science*, *311*(5764), 1123-1127.

Ji, Q., Luo, Z. X., Yuan, C. X., Wible, J. R., Zhang, J. P., & Georgi, J. A. (2002). The earliest known eutherian mammal. *Nature*, *416*(6883), 816-822.

Ji, Q., Luo, Z. X., Zhang, X., Yuan, C. X., & Xu, L. (2009). Evolutionary development of the middle ear in Mesozoic therian mammals. *Science*, *326*(5950), 278-281.

Kielan-Jaworowska, Z., Cifelli, R. L., & Luo, Z. X. (2004). *Mammals from the age of dinosaurs: origins, evolution, and structure*. Columbia University Press, New York, NY.

Kielan-Jaworowska, Z., & Dashzeveg, D. (1989). Eutherian mammals from the Early Cretaceous of Mongolia. *Zoologica Scripta*, *18*(2), 347-355.

Krause, D. W., et al. (2014). First cranial remains of a gondwanatherian mammal reveal remarkable mosaicism. *Nature*, *515*(7528), 512-517.

Krebs, B. (1991). Das Skelett von Henkelotherium guimarotae gen. et sp. nov.(Eupantotheria, Mammalia) aus dem Oberen Jura von Portugal. *Selbstverlag Fachbereich Geowissenschaften, 133,* 1-110.

Krebs, B. (1993). Das Gebiss von Crusafontia (Eupantotheria, Mammalia)–Funde aus der Unter− Kreide von Galve und Uña (Spanien). *Berliner geowissenschaftliche Abhandlungen E*, *9*, 233-252.

Kusuhashi, N., Tsutsumi, Y., Saegusa, H., Horie, K., Ikeda, T., Yokoyama, K., & Shiraishi, K. (2013). A new Early Cretaceous eutherian mammal from the Sasayama Group, Hyogo, Japan. *Proceedings of the Royal Society of London B: Biological Sciences*, *280*(1759), 20130142.

Labandeira, C. C., Johnson, K. R., & Wilf, P. (2002). Impact of the terminal Cretaceous event on plant–insect associations. *Proceedings of the National Academy of Sciences*, *99*(4), 2061-2066.

Law, C. J., Young, C., & Mehta, R. S. (2016). Ontogenetic scaling of theoretical bite force in southern sea otters (*Enhydra lutris nereis*). *Physiological and Biochemical Zoology*, *89*(5), 347-363.

Lillegraven, J. A., & Krusat, G. (1991). Cranio-mandibular anatomy of Haldanodon exspectatus (Docodonta; Mammalia) from the Late Jurassic of Portugal and its implications to the evolution of mammalian characters.*Rocky Mountain Geology*, *28*(2), 39-138.

Lloyd, G. T., et al. (2008). Dinosaurs and the Cretaceous terrestrial revolution. *Proceedings of the Royal Society of London B: Biological Sciences*,*275*(1650), 2483-2490.

Lopatin, A. V., Maschenko, E. N., Averianov, A. O., Rezvyi, A. S., Skutschas, P. P., & Leshchinskiy, S. V. (2005). Early Cretaceous mammals from Western Siberia: 1. Tinodontidae. *Paleontological Journal*, *39*(5), 523.

Luo, Z. X. (2007). Transformation and diversification in early mammal evolution. *Nature*, *450*(7172), 1011-1019.

Luo, Z. X., Chen, P., Li, G., & Chen, M. (2007A). A new eutriconodont mammal and evolutionary development in early mammals. *Nature*, *446*(7133), 288-293.

Luo, Z. X., Crompton, A. W., & Sun, A. L. (2001). A new mammaliaform from the early Jurassic and evolution of mammalian characteristics. *Science*, *292*(5521), 1535-1540.

Luo, Z. X., Ji, Q., Wible, J. R., & Yuan, C. X. (2003). An Early Cretaceous tribosphenic mammal and metatherian evolution. *Science*, *302*(5652), 1934-1940.

Luo, Z. X., Ji, Q., & Yuan, C. X. (2007B). Convergent dental adaptations in pseudo-tribosphenic and tribosphenic mammals. *Nature*, *450*(7166), 93-97.

Luo, Z. X., Gatesy, S. M., Jenkins, F. A., Amaral, W. W., & Shubin, N. H. (2015A). Mandibular and dental characteristics of Late Triassic mammaliaform Haramiyavia and their ramifications for basal mammal evolution. *Proceedings of the National Academy of Sciences*, *112*(51), E7101-E7109.

Luo, Z. X., Kielan-Jaworowska, Z., & Cifelli, R. L. (2002). In quest for a phylogeny of Mesozoic mammals. *Acta Palaeontologica Polonica*, *47*(1), 1-78.

Luo, Z. X., & Martin, T. (2007). Analysis of molar structure and phylogeny of docodont genera. *Bulletin of Carnegie Museum of Natural History*, 27-47.

Luo, Z. X., Meng, Q. J., Ji, Q., Liu, D., Zhang, Y. G., & Neander, A. I. (2015B). Evolutionary development in basal mammaliaforms as revealed by a docodontan. *Science*, *347*(6223), 760-764.

Luo, Z. X., & Wible, J. R. (2005). A Late Jurassic digging mammal and early mammalian diversification. *Science*, *308*(5718), 103-107.

Luo, Z. X. (2011). Developmental patterns in Mesozoic evolution of mammal ears. *Annual Review of Ecology, Evolution, and Systematics*, *42*, 355-380.

Luo, Z. X., Yuan, C. X., Meng, Q. J., & Ji, Q. (2011). A Jurassic eutherian mammal and divergence of marsupials and placentals. *Nature*, *476*(7361), 442-445.

Martin, T. (1999). Dryolestidae (Dryolestoidea, Mammalia) aus dem Oberen Jura von Portugal. *Abhandlungen der Senckenbergischen Naturforschenden Gesellschaft, 550,* 1-119.

Martin, T., Marugán-Lobón, J., Vullo, R., Martín-Abad, H., Luo, Z. X., & Buscalioni, A. D. (2015). A Cretaceous eutriconodont and integument evolution in early mammals. *Nature*, *526*(7573), 380-384.

Maynard Smith, J., & Savage, R. J. G. (1959). The mechanics of mammalian jaws. *School Science Review*, *141*, 289-301.

McKenna, M. C. (1975). Toward a phylogenetic classification of the Mammalia. In Luckett, W. P., & Szalay, F. S. (eds) *Phylogeny of the Primates*, 21-46. Plenum Press, New York.

Meng, J., Hu, Y., Wang, Y., Wang, X., & Li, C. (2006). A Mesozoic gliding mammal from northeastern China. *Nature*, *444*(7121), 889-893.

Meng, J., Wang, Y., & Li, C. (2011). Transitional mammalian middle ear from a new Cretaceous Jehol eutriconodont. *Nature*, *472*(7342), 181-185.

Meng, Q. J., Ji, Q., Zhang, Y. G., Liu, D., Grossnickle, D. M., & Luo, Z. X. (2015). An arboreal docodont from the Jurassic and mammaliaform ecological diversification. *Science*, *347*(6223), 764-768.

O'Meara, R. N., & Thompson, R. S. (2014). Were there miocene meridiolestidans? Assessing the phylogenetic placement of necrolestes patagonensis and the presence of a 40 million year meridiolestidan ghost lineage. *Journal of Mammalian Evolution*, *21*(3), 271-284.

Pfretzschner, H. U., Martin, T., Matzke, A. T., & Sun, G. (2005). A new docodont mammal from the Late Jurassic of the Junggar Basin in Northwest China. *Acta Palaeontologica Polonica*, *50*(4), 799-808.

Polly, P. D. (2016). *Geometric morphometrics for Mathematica*. Version 12.0. Department of Geological Sciences, Indiana University, Bloomington, IN.

Prothero, D. R. (1981). New jurassic mammals from Como Bluff, Wyoming and the interrelationships of nontribosphenic Theria. *Bulletin of the American Museum of Natural History,* 57, 1040–1046.

R Core Team (2016). R: a language and environment for statistical computing. R Foundation for Statistical Computing, Vienna. http://www.R-project.org/.

Ramírez-Chaves, H. E., Weisbecker, V., Wroe, S., & Phillips, M. J. (2016). Resolving the evolution of the mammalian middle ear using Bayesian inference. *Frontiers in Zoology*, *13*(1), 39.

Rauhut, O. W., Martin, T., Ortiz-Jaureguizar, E., & Puerta, P. (2002). A Jurassic mammal from South America. *Nature*, *416*(6877), 165-168.

Reed, D. A., Iriarte-Diaz, J., & Diekwisch, T. G. H. (2016). A three dimensional free body analysis describing variation in the musculoskeletal configuration of the cynodont lower jaw. *Evolution & development*, *18*(1), 41-53.

Rich, T. H., Flannery, T. F., Trusler, P., Kool, L., Van Klaveren, N. A., & Vickers-Rich, P. (2002). Evidence that monotremes and ausktribosphenids are not sistergroups. *Journal of Vertebrate Paleontology*, *22*(2), 466-469.

Rich, T., Flannery, T., Trusler, P., Kool, L., Van Klaurer, N., & Vickers-Rich, P. (2001). A second tribosphenic mammal from the Mesozoic of Australia. *Records of the Queen Victoria Museum*, *110*, 1-9.

Rich, T. H., et al. (2016). The mandible and dentition of the Early Cretaceous monotreme Teinolophos trusleri. *Alcheringa: An Australasian Journal of Palaeontology*, 1-27.

Rich, T. H., Vickers-Rich, P., Constantine, A., Flannery, T. F., Kool, L., & van Klaveren, N. (1997). A tribosphenic mammal from the Mesozoic of Australia. *Science*, *278*(5342), 1438-1442.

Rougier, G. W. (1993). Vincelestes neuquenianus Bonaparte (Mammalia, Theria) un primitivo mamífero del Cretácico Inferior de la Cuenca Neuquina.*Unpublished Ph. D. dissertation*.

Rougier, G. W., Apesteguía, S., & Gaetano, L. C. (2011). Highly specialized mammalian skulls from the Late Cretaceous of South America. *Nature*, *479*(7371), 98-102.

Rougier, G. W., Martinelli, A. G., Forasiepi, A. M., & Novacek, M. J. (2007). New Jurassic mammals from Patagonia, Argentina: a reappraisal of australosphenidan morphology and interrelationships. *American Museum Novitates, 3566*, 1-54.

Rougier, G. W., Wible, J. R., Beck, R. M., & Apesteguía, S. (2012). The Miocene mammal Necrolestes demonstrates the survival of a Mesozoic nontherian lineage into the late Cenozoic of South America. *Proceedings of the National Academy of Sciences*, *109*(49), 20053-20058.

Rougier, G. W., Sheth, A. S., Carpenter, K., Appella-Guiscafre, L., & Davis, B. M. (2015). A new species of docodon (Mammaliaformes: Docodonta) from the Upper Jurassic Morrison Formation and a reassessment of selected craniodental characters in basal mammaliaforms. *Journal of Mammalian Evolution*, *22*(1), 1-16.

Simpson, G. G. (1925). Mesozoic Mammalia, II; Tinodon and its allies. *American Journal of Science*, *10,* 451-470.

Simpson, G. G. (1928). *A catalogue of the Mesozoic Mammalia in the Geological Department of the British Museum*. Trustees of the British Museum, London.

Simpson, G. G. (1929). American Mesozoic Mammalia. *Memoirs of the Peabody Museum of Yale University, 3*, 1-235.

Simpson, G. G. (1944). *Tempo and mode in evolution*. Columbia University Press.

Smits, P. D. (2015). Expected time-invariant effects of biological traits on mammal species duration. *Proceedings of the National Academy of Sciences*, *112*(42), 13015-13020.

Tobin, T. S., et al. (2012). Extinction patterns, δ^{18} O trends, and magnetostratigraphy from a southern high-latitude Cretaceous–Paleogene section: Links with Deccan volcanism. *Palaeogeography, Palaeoclimatology, Palaeoecology*, *350*, 180-188.

Turnbull, W. D. (1970). Mammalian masticatory apparatus. *Fieldiana: Geology*, *18*, 147-356.

Wang, Y., Hu, Y., Meng, J., & Li, C. (2001). An ossified Meckel's cartilage in two Cretaceous mammals and origin of the mammalian middle ear. *Science*, *294*(5541), 357-361.

Yuan, C. X., Ji, Q., Meng, Q. J., Tabrum, A. R., & Luo, Z. X. (2013). Earliest evolution of multituberculate mammals revealed by a new Jurassic fossil. *Science*, *341*(6147), 779-783.

Wall, C. E., & Krause, D. W. (1992). A biomechanical analysis of the masticatory apparatus of Ptilodus (Multituberculata). *Journal of Vertebrate Paleontology*, *12*(2), 172-187.

Weijs, W. A. (1994). Evolutionary approach of masticatory motor patterns in mammals. In *Biomechanics of feeding in vertebrates*, 281-320. Springer Berlin Heidelberg.

Williams, S. H., Vinyard, C. J., Wall, C. E., Doherty, A. H., Crompton, A. W., & Hylander, W. L. (2011). A preliminary analysis of correlated evolution in mammalian chewing motor patterns. *Integrative and comparative biology*, *51*(2), 247-259.

Wilson, G. P. (2014). Mammalian extinction, survival, and recovery dynamics across the Cretaceous-Paleogene boundary in northeastern Montana, USA. *Geological Society of America Special Papers*, *503*, 365-392.

Wilson, G. P., Evans, A. R., Corfe, I. J., Smits, P. D., Fortelius, M., & Jernvall, J. (2012). Adaptive radiation of multituberculate mammals before the extinction of dinosaurs. *Nature*, *483*(7390), 457-460.

Zhang, F., Crompton, A. W., Luo, Z., & Schaff, C. R. (1998). Pattern of dental replacement of Sinoconodon and its implication for evolution of mammals. *Vertebrata Pal Asiatica*, *36*(3), 197-217.

Zheng, X., Bi, S., Wang, X., & Meng, J. (2013). A new arboreal haramiyid shows the diversity of crown mammals in the Jurassic period. *Nature*, *500*(7461), 199-202.

Zhou, C. F., Wu, S., Martin, T., & Luo, Z. X. (2013). A Jurassic mammaliaform and the earliest mammalian evolutionary adaptations. *Nature*, *500*(7461), 163-167.
